# Eumelanin and pheomelanin are predominant pigments in bumblebee (Apidae: *Bombus*) pubescence

**DOI:** 10.7717/peerj.3300

**Published:** 2017-05-24

**Authors:** Carlo Polidori, Alberto Jorge, Concepción Ornosa

**Affiliations:** 1 Instituto de Ciencias Ambientales, Universidad de Castilla La Mancha, Toledo, Spain; 2 Laboratorio de Microscopía, Museo Nacional de Ciencias Naturales (CSIC), Madrid, Spain; 3 Departamento de Zoología y Antropología Física, Universidad Complutense de Madrid, Madrid, Spain

**Keywords:** *Bombus*, Eumelanin, Depigmentation, Pigmentation, Pheomelanin, Pubescence, Phenotype, Hymenoptera, Raman spectroscopy

## Abstract

**Background:**

Bumblebees (Hymenoptera: Apidae: *Bombus*) are well known for their important inter- and intra-specific variation in hair (or pubescence) color patterns, but the chemical nature of the pigments associated with these patterns is not fully understood. For example, though melanization is believed to provide darker colors, it still unknown which types of melanin are responsible for each color, and no conclusive data are available for the lighter colors, including white.

**Methods:**

By using dispersive Raman spectroscopy analysis on 12 species/subspecies of bumblebees from seven subgenera, we tested the hypothesis that eumelanin and pheomelanin, the two main melanin types occurring in animals, are largely responsible for bumblebee pubescence coloration.

**Results:**

Eumelanin and pheomelanin occur in bumblebee pubescence. Black pigmentation is due to prevalent eumelanin, with visible signals of additional pheomelanin, while the yellow, orange, red and brown hairs clearly include pheomelanin. On the other hand, white hairs reward very weak Raman signals, suggesting that they are depigmented. Additional non-melanic pigments in yellow hair cannot be excluded but need other techniques to be detected. Raman spectra were more similar across similarly colored hairs, with no apparent effect of phylogeny and both melanin types appeared to be already used at the beginning of bumblebee radiation.

**Discussion:**

We suggest that the two main melanin forms, at variable amounts and/or vibrational states, are sufficient in giving almost the whole color range of bumblebee pubescence, allowing these insects to use a single precursor instead of synthesizing a variety of chemically different pigments. This would agree with commonly seen color interchanges between body segments across *Bombus* species.

## Introduction

One of the distinct features of animal phenotypic variation is certainly coloration, and in many cases it depends on pigments incorporated in cells and tissues (Bennett & Thery, 2007), with melanins being probably the most prevalent ones (Riley, 1997; Nosanchuk & Casadevall, 2006; Gao & Garcia-Pichel, 2011; Eisenman & Casadevall, 2012; Eliason, Bitton & Shawkey, 2013). In insects, for example, a great variability in pigmentation pattern can be found among species, among and within populations and across different life-stages (Majerus, 1998; Wittkopp & Beldade, 2009), and it depends in many cases on type and degree of melanization (Needham, 1978; Kohler et al., 2007; Lindstedt et al., 2010; Shamim et al., 2014). Melanins protect cells from the damaging effects of ultraviolet (UV) radiation (Brenner & Hearing, 2008; Gao & Garcia-Pichel, 2011) and are also involved in thermoregulation, immune response, cuticle hardening, desiccation resistance and both intra- and inter-specific visual communication (Cerenius & Söderhäll, 2004; Nappi & Christensen, 2005; Dubovskiy et al., 2013; Różanowska et al., 1999; Riley, 1997; Ramniwas et al., 2013; Searcy & Nowicki, 2005; Price et al., 2008; Koch, Behnecke & ffrench-Constant, 2000; Galván & Møller, 2013).

Melanins are heterogeneous metabolites composed by polyphenolic compounds and derived from the oxidative condensation of the aminoacid l-tyrosine through enzymatic reactions involving tyrosinase, the key enzyme of melanogenesis in animals (Ito et al., 2011; García-Borrón & Olivares Sánchez, 2011). One of the intermediate in the biosynthesis of melanin is dopachrome, which is then polymerized into various forms of melanin, including eumelanin and pheomelanin, which are the most widespread in nature. Eumelanin is a polymer of indole units produced via formation of 5,6-dihydroxyindole (DHI) and 5,6-dihydroxyindole-2-carboxylic acid (DHICA), and is produced from intramolecular cyclization of dopaquinone in the absence of thiol compounds; this pigment generally leads to dark colorations (García-Borrón & Olivares Sánchez, 2011). Pheomenalin, also deriving from dopaquinone, is an oligomer of sulfur-containing heterocycles (benzothiazine and benzothiazole) produced with the intervention of thiols, such as l-cysteine; this pigment generally leads to yellowish to brownish coloration (Riddle, 1909; Simon & Peles, 2010; García-Borrón & Olivares Sánchez, 2011).

Though both types of melanin were frequently detected in vertebrates (Ito & Wakamatsu, 2003; Roulin, Mafli & Wakamatsu, 2013; Wolnicka-Glubisz et al., 2012), only eumelanin was known to be widespread in invertebrates (Needham, 1978; Kohler et al., 2007; Lindstedt et al., 2010; Shamim et al., 2014). Indeed, pheomelanin has been detected very recently only in one mollusk (Speiser, DeMartini & Oakley, 2014) and four insect species (a grasshopper, a butterfly and two parasitoid wasps) (Galván et al., 2015; Jorge García, Polidori & Nieves-Aldrey, 2016; Wakamatsu et al., unpublished data cited in Galván et al., 2015). These recent findings suggest that pheomelanin could be more frequent in insects than previously believed, and bees (Apoidea) are good models to check for its presence given their extremely variable colors, spanning from black to red, yellow, orange and brown (Michener, 2007).

One bee genus that particularly shows huge variation in color patterns is *Bombus* Latreille, 1802 (bumblebees). Indeed, the bumblebee body is covered by a dense layer of hair-like extensions of the cuticle, known as pubescence (Heinrich, 2004; Williams, 2007; Hines & Williams, 2012; Rapti, Duennes & Cameron, 2014). The diversity in bumblebee color patterns is due to the variation in the color of the pubescence, since the underlying cuticle of bumblebee is black. Differences and similarities in color patterns are well known among and within bumblebee species (Dalla Torre, 1880; Vogt, 1911; Rapti, Duennes & Cameron, 2014), and the diverse, segment-specific patterns seem to have evolved early in the genus history (Cameron, Hines & Williams, 2007). These observations lead to different hypotheses on the adaptive role of color pattern diversification and convergence in these insects, including thermoregulation (Heinrich, 2004), aposematism/Mullerian mimicry (Evans & Waldbauer, 1982; Plowright & Owen, 1980) and crypsis (Williams, 2007).

All these efforts in studying the variability and evolution of color patterns in bumblebees contrast, however, with the still unclear evidence on their chemical nature. As far as we know, indeed, previous work based on different analytical tools such as thin layer chromatography (TLC), Spectrophotometry and HPLC/mass spectrometry (MS) suggested black, orange, brown and red coloration depending on melanin (without further distinction of melanin types) (Babiy, 1925; Owen & Plowright, 1980; Hines, 2008a). On the other hand, the pigment responsible for yellow color in bumblebees was not determined in detail yet but it was suggested to be possibly a pterin (Hines, 2008a). Furthermore, it is actually unknown whether white is due to a pigment or to the absence of pigments (Hines, 2008a).

Here, by using dispersive Raman spectroscopy analysis, we aimed to test which types of melanin occur in bumblebee hairs of different colors. We show for the first time that eumelanin occurs in black hair and pheomelanin in hairs of all the other colors. The only exception is white, which seems to be due to depigmentation.

## Methods

### Study organisms

**Figure 1:**
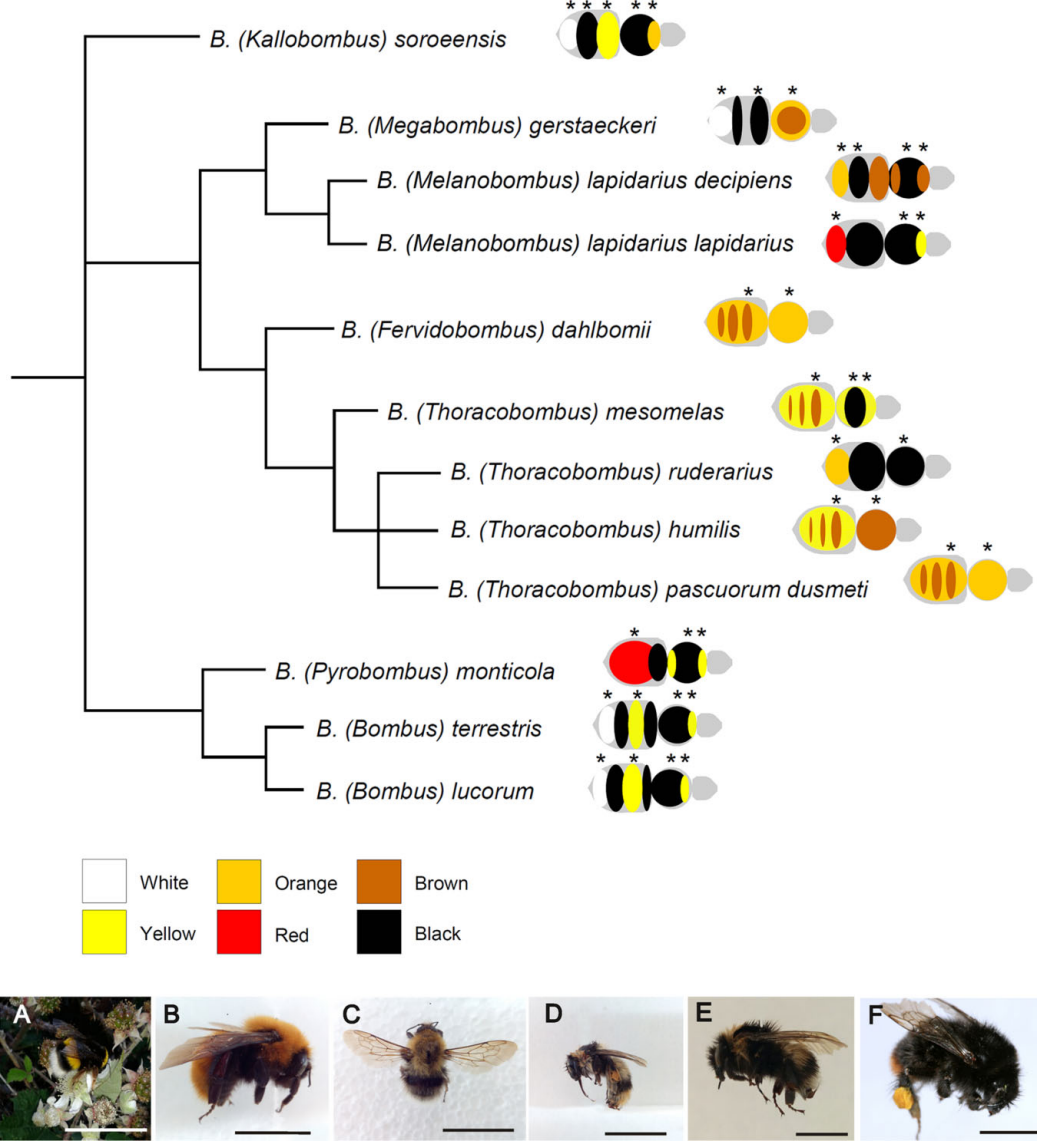
Phylogenetic relationships among the studied species of *Bombus*, hand-drawn starting from the results published in Cameron, Hines & Williams, 2007. Close to each species/subspecies one finds the simplified color pattern of the pubescence, as observed in the studied individuals; the analyzed areas are indicated by asterisks. The range of colors and corresponding nomenclature is shown below the tree. Representative pictures of some of the studied species/subspecies are at the bottom of the figure: (A) *Bombus terrestris*; (B) *Bombus dahlbomii*; (C) *Bombus gerstaerckeri*; (D) *Bombus lapidarius decipiens*; (E) *Bombus soroeensis*; (F) *Bombus lapidarius lapidarius* (bar = 1 cm).

Females (either queens or workers, or both) of 11 species (one represented by two subspecies) of *Bombus* from seven subgenera were selected for the study: *Bombus (Bombus) lucorum* (Linnaeus, 1761), *Bombus (Bombus) terrestris* Linnaeus, 1758, *Bombus (Fervidobombus) dahlbomii* Guerin-Meneville, 1835—*Bombus (Kallobombus) soroeensis* (Fabricius, 1777), *Bombus (Megabombus) gerstaeckeri* Morawitz, 1881, *Bombus (Melanobombus) lapidarius decipiens* Pérez, 1890, *Bombus (Melanobombus) lapidarius lapidarius* (Linnaeus,1758), *Bombus (Pyrobombus) monticola* Smith, 1849, *Bombus (Thoracobombus) humilis* Illiger, 1806, *Bombus (Thoracobombus) mesomelas* Gerstaecker, 1869, *Bombus (Thoracobombus) pascuorum dusmeti* Vogt, 1909 and *Bombus (Thoracobombus) ruderarius* (Müller, 1776) (Fig. 1). All individuals were collected by netting on flowers in various years and at various localities in Spain and Chile (Ornosa & Ortiz-Sánchez, 2004; Polidori et al., 2014), killed by freezing and then deposited in public or private collection as pinned dry specimen (Table 1). Field collections were approved by the Ministerio de Medio Ambiente y Medio Rural y Marino (Spain) (permit number: CO/09/106/2010), Comunidad de Madrid (Spain) (permit number: 10/091686.9/15), Junta de Castilla y León (Spain) (permit number: EP/CYL/366/2013), Gobierno de Aragón (Spain) (permit number: CSVM5-1XA8S-75HA5-OUREG), Gobierno de Navarra (granted on 25/05/2009) and Estación Cientifica de Huinay (Chile) (granted on 18/10/2014). On the whole, 46 individuals (30 workers and 16 queens) were analyzed, three to four per species/subspecies (Table 1). In our sample, color patterns were very similar within species between queens and workers (Fig. S1), so that no further distinction between castes was done during the analysis.

**Table 1:** Details of the data collection of bumblebees (*Bombus* spp.) used in this study.

Subgenus	Species	*N*	Geographic origin	Collection
*Bombus*	*Bombus lucorum*	4 W	Huesca (Spain)	UCME
*Bombus*	*Bombus terrestris*	4 Q	Madrid, Badajoz, Pontevedra (Spain)	UCME
*Fervidobombus*	*Bombus dahlbomii*	4 W	Huinay, Hualaihué (Chile)	MNCN
*Kallobombus*	*Bombus soroeensis*	4 W	León (Spain)	UCME
*Megabombus*	*Bombus gerstaeckeri*	3 W	León, Huesca (Spain)	UCME
*Melanobombus*	*Bombus lapidarius decipiens*	2 Q, 1 W	León (Spain)	UCME
*Melanobombus*	*Bombus lapidarius lapidarius*	2 Q, 2 W	León, Navarra, Huesca (Spain)	UCME
*Pyrobombus*	*Bombus monticola*	4 W	Cantabria (Spain)	UCME
*Thoracobombus*	*Bombus humilis*	3 Q, 1 W	León, Huesca (Spain)	UCME
*Thoracobombus*	*Bombus mesomelas*	4 W	León (Spain)	UCME
*Thoracobombus*	*Bombus pascuorum dusmeti*	4 Q	Madrid (Spain)	UCME
*Thoracobombus*	*Bombus ruderarius*	1 Q, 3 W	Huesca (Spain)	UCME

**Note:**

W, worker; Q, queen; UCME, Museo de Entomología de la Universidad Complutense de Madrid; MNCN, Museo Nacional de Ciencias Naturales (CSIC).

### Analysis of pigments

To analyze if melanins are responsible for the pigmentation of bumblebee hair we used dispersive Raman spectroscopy, which was proved to be a useful non-destructive technique to analyze these pigments (Galván et al., 2013; Galván & Jorge, 2015; Jorge García, Polidori & Nieves-Aldrey, 2016), known to be almost completely insoluble in all solvents (Gonçalves, Lisboa & Pombeiro-Sponchiado, 2012). The Raman analysis produces spectra in which signatures of prevalent molecules are visible, allowing their identification through comparisons with published spectra and databases (Colthub, Daly & Wiberley, 1990; Czamara et al., 2015; Galván & Jorge, 2015). We introduced an intact individual into a Thermo Fisher DXR confocal dispersive Raman microscope (Thermo Fisher Scientific, Madison, WI, USA), which was associated with Thermo Fisher OMNIC 8.1 software. For each species, we checked for the occurrence of eumelanin and pheomelanin in differently colored hairs, as detailed in Fig. 1, by point-and-shoot analysis. The spatial resolution was 1 μm and the excitation laser source was at 780 nm of 2–7 mW power, which are optimal parameters for melanin detection in Raman (Galván & Jorge, 2015; Jorge García, Polidori & Nieves-Aldrey, 2016). A 50× confocal objective, a slit aperture of 25 μm and a grating of 400 lines/mm were used to obtain the spectra, which had an average resolution of 2.2–4.4 cm^−1^ in the wavenumber range of 150–2,500 cm^−1^. The typical measured linewidth (FWHH) of an average of four spectra in two bands of polystyrene centered at 1002.30 and 1603.06 were 6.2 cm^−1^ and 8.9 cm^−1^, respectively. An integration time of 5 s × 12 accumulations allowed getting an acceptable signal to noise ratio. Pure polystyrene was used to check calibration and aligning of the spectra.

We described the color of each analyzed pubescence area as white, yellow, orange, orange–red/red (red in the following text), orange–brown/brown (brown in the following text) or black. For any given individual and pigmented area, the analysis was repeated four times at different points. We also analyzed for one species (*Bombus terrestris*) the black ventral side of the thorax, which lacks hairs.

Because the yellow pigment in bumblebees was previously suggested to be due to non-melanic pigments (Babiy, 1925) and probably to a pterin (Hines, 2008a) we performed two additional experiments. First, we carried out the Raman analysis on hair-extracted yellow pigment from 20 workers of one species (*Bombus terrestris*, one of those with the brightest yellow). Extraction of the yellow pigment from hairs (200 mg on fresh weight) was carried out through acid MeOH and NaHCO_3_ 0.01 M following Hines (2008a), which used the extracts for chemical characterization with TLC and HPLC/MS, and the extracts were then moved to the Raman microscope. Second, we carried out the Raman analysis on commercially available synthetic 95% solid pterin on KBr disk obtained from Sigma-Aldrich, Saint Louis, MO, USA (P1132). Conditions of these Raman analyses were the same as described above.

A visual comparison with spectra retrieved by recent literature and reference database allowed their association with eumelanin or pheomelanin (Galván et al., 2015; Galván & Jorge, 2015; Huang et al., 2004; De Gelder et al., 2007). Peaks known to be associated with chitin, the fundamental polysaccharide composing the insect cuticle (De Gelder et al., 2007), and its precursor *N*-acetyl-d-glucosamine, the structural monomer of chitin (Jamialahmadi et al., 2011), were also identified by visual inspection. Then, to improve the main peak identification, the Raman spectra were analyzed by the reference deconvolution method (Middendorf, 1974; Morris, Barjat & Horne, 1997) as in Jorge García, Polidori & Nieves-Aldrey (2016), using the software ORIGIN v.7 (OriginLab Corporation, Northampton, MA, USA). This method circumvents distortions affecting the spectral peaks by using a well-known reference signal, and then reconvolutes the spectrum with a Lorentzian lineshape (Middendorf, 1974).

The statistical analysis was carried out on the mean Raman intensity values calculated across individuals for each species/body part/color, giving a total of 47 spectra (data available as Supplemental Data). To explore the dissimilarities among the differently pigmented areas and the different species/subspecies based on Raman spectra we used two methods. First, we performed an agglomerative hierarchical clustering (AHC) (Gordon, 1999). This method found relatively homogeneous clusters of cases based on Raman peak values (each case represented by the mean peak values across individuals for a given color, body part and species), and it was performed through Ward’s method based on Euclidean distance (dissimilarity) between pairs of objects. The analysis also provides the dissimilarity value that discriminates the major different clusters. Then, using the same Euclidean dissimilarity matrix, we performed a classical multidimensional scaling analysis (Torgerson, 1952) for a bi-plot representation of the distribution of the cases in the six color categories. This technique is commonly used to determine a *n*-dimensional space and corresponding coordinates for a set of objects, using a single matrix of pair wise dissimilarities between these objects (Borg & Groenen, 2005). The cluster analysis and the multidimensional scaling analysis were carried out in the software XlStat 2012 (Addinsoft, New York, NY, USA).

## Results

The studied *Bombus* species/subspecies presented a variety of color patterns (Fig. 1). Some, like *Bombus terrestris* and *Bombus lucorum*, present a combination of black, yellow and white; others, like *Bombus monticola* and *Bombus lapidarius lapidarius*, present a combination of yellow, red and black; other species (e.g., *Bombus dahlbomii* and *Bombus pascuorum dusmeti*) present exclusively an orange/brown pubescence (Fig. 1).

The inspection of Raman spectra revealed that all the colorations found in the bumblebee hairs largely depend on the occurrence of eumelanin and/or pheomelanin, or on the lack of both pigments.

**Figure 2:**
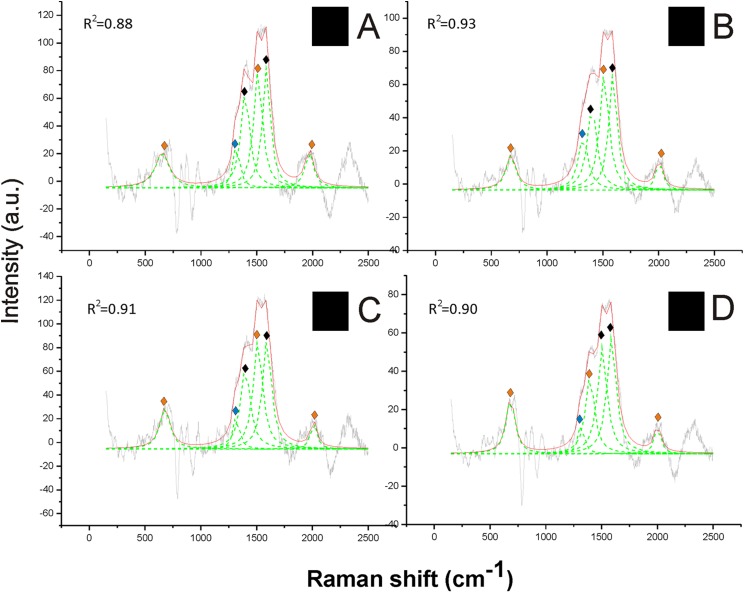
Examples of Raman spectra of black hair in *Bombus* and peak identification after having applied the reference deconvolution method. The gray line represents the Raman spectrum, the dashed green lines represent the single deconvoluted curves, which highlight the different peaks contributing to the spectrum, and the red line represents the sum of the deconvoluted curves (i.e., the adjustment to the spectrum, whose goodness of fit expressed as *R*^2^ value). ♦ Signature peaks for eumelanin, 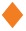 signature peaks for pheomelanin, 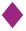 signature peaks for chitin. (A) Thorax of *Bombus lucorum*; (B) thorax of *Bombus monticola*; (C) abdomen of *Bombus soroeensis*; (D) thorax of *Bombus terrestris*.

The Raman spectra revealed a strong signal of eumelanin, with its typical signature showing two main bands at about 1,385 cm^−1^ and at about 1,580 cm^−1^, in the black hair (Fig. 2). These two peaks were well highlighted through the reference deconvolution method (Fig. 2). These spectra clearly resembled eumelanin spectra known for other organisms (Huang et al., 2004; Perna et al., 2013; Centeno & Shamir, 2008) as well as the spectrum of synthetic eumelanin (Kim et al., 2015). Major peaks were associated with the vibration of stretching of the hexagonal carbon rings in the molecule structure, the vibration of three of the six C–C bounds within the rings and the vibration of the C–H of methyl and methylene groups in the eumelanin polymers (Huang et al., 2004).

Two further peaks at about 1,490 and 2,000 cm^−1^ from black hair spectra can be associated with the additional presence of pheomelanin (Fig. 2). These two peaks were found associated with synthetic pheomelanin spectrum (Kim et al., 2015) as well as with pheomelanin spectra in other organisms (Galván et al., 2015). These peaks have been assigned, respectively, by the out-of-plane deformation and the stretching vibration of the phenyl rings in the molecule structure, and to overtone or combination bands (Galván et al., 2013). Thus, black hair seems to include both melanin types.

**Figure 3:**
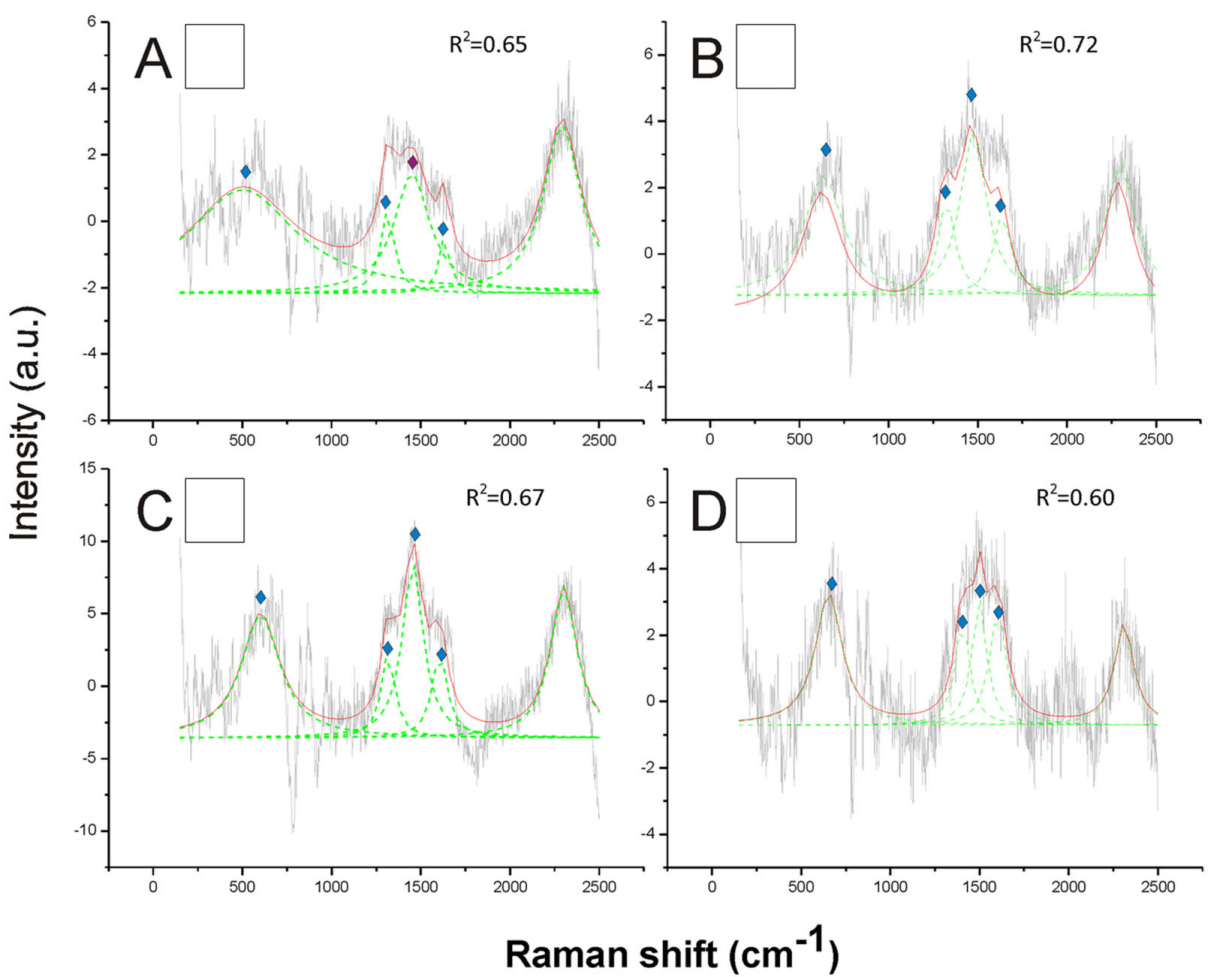
Examples of Raman spectra of white hair, and of hairless thorax ventral side cuticle, in *Bombus*, and peak identification after having applied the reference deconvolution method. The gray line represents the Raman spectrum, the dashed green lines represent the single deconvoluted curves, which highlight the different peaks contributing to the spectrum, and the red line represents the sum of the deconvoluted curves (i.e., the adjustment to the spectrum, whose goodness of fit expressed as *R*^2^ value). 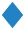 Signature peaks for chitin, 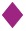 signature peaks for *N*-acetyl-d-glucosamine. (A) abdomen of *Bombus lucorum*; (B) abdomen of *Bombus terrestris*; (C) abdomen of *Bombus soroeensis*; (D) abdomen of *Bombus gerstaeckeri.* Note that no melanin peaks and overall very low intensity signal were detected in white hair.

Opposite to black, the white hair appeared to be due to the absence of any type of melanin (Fig. 3). These spectra showed a very low Raman intensity signal (maximum about 5 AU), particularly when compared with most of the spectra obtained for all the other colors (maximum between about 30 and 150 AU), and they are clearly noisy in their patterns (Fig. 3). The reference deconvolution method confirms that no peaks can be associated with either eumelanin or pheomelanin, though it recognized peaks associated with chitin (see below) (Fig. 3).

**Figure 4:**
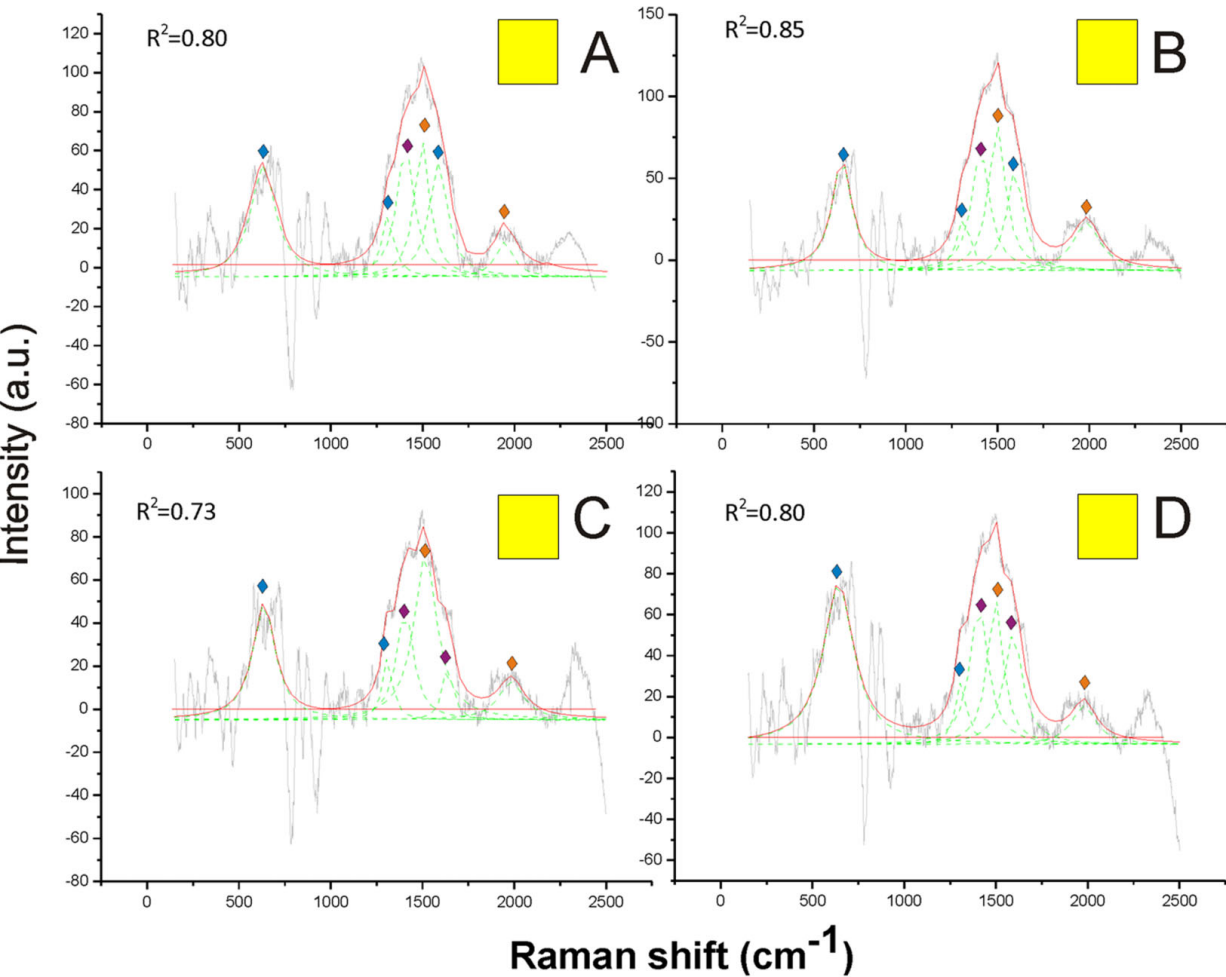
Examples of Raman spectra of yellow hair in *Bombus*, and peak identification after having applied the reference deconvolution method. The gray line represents the Raman spectrum, the dashed green lines represent the single deconvoluted curves, which highlight the different peaks contributing to the spectrum, and the red line represents the sum of the deconvoluted curves (i.e., the adjustment to the spectrum, whose goodness of fit expressed as *R*^2^ value). 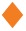 Signature peaks for pheomelanin, 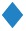 signature peaks for chitin, 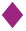 signature peaks for *N*-acetyl-d-glucosamine. (A) Abdomen of *Bombus soroeensis*; (B) abdomen of *Bombus terrestris*; (C) thorax of *Bombus lucorum*; (D) thorax of *Bombus monticola*.

**Figure 5:**
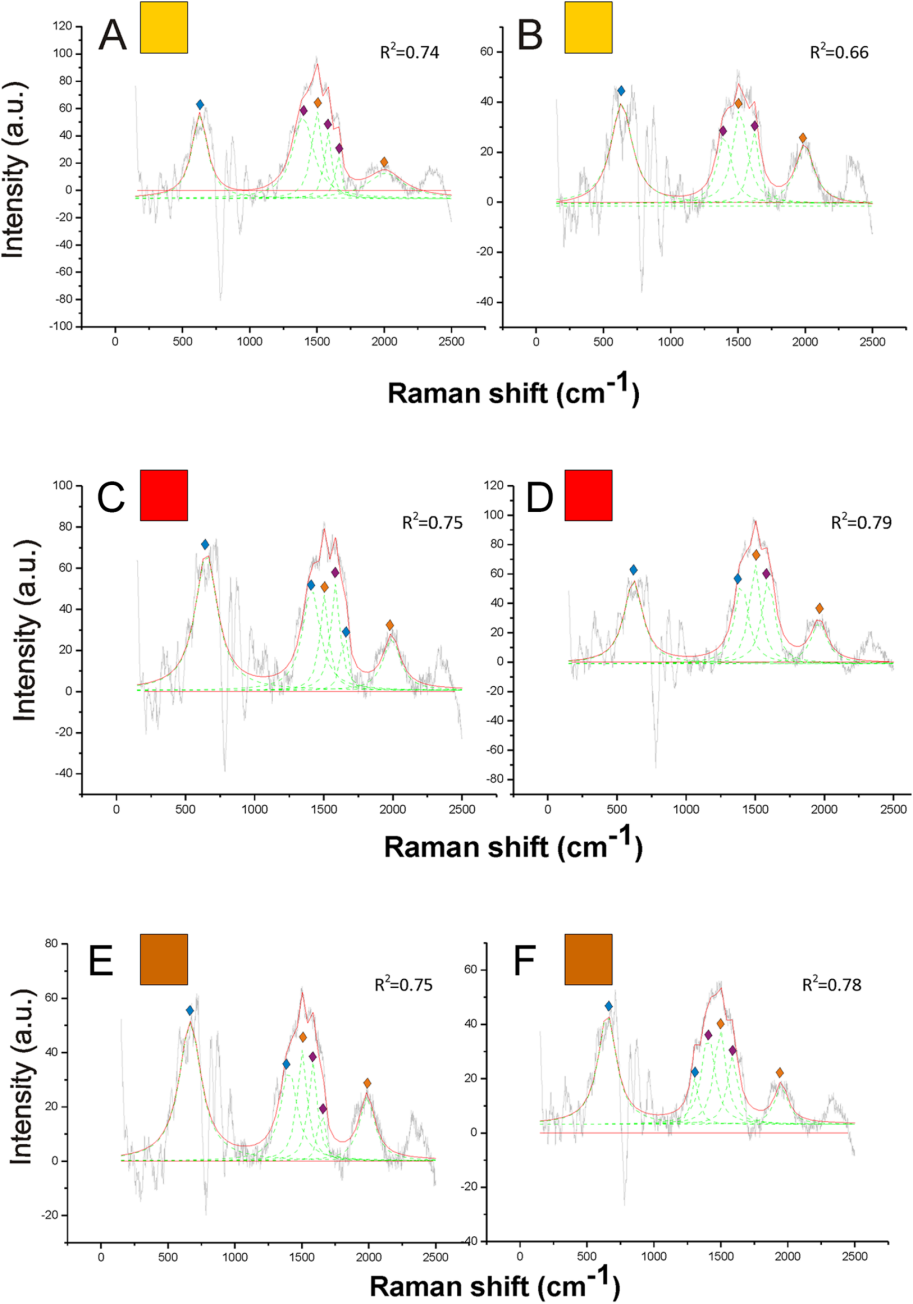
Examples of Raman spectra of orange, red and brown hair in *Bombus*, and peak identification after having applied the reference deconvolution method. The gray line represents the Raman spectrum, the dashed green lines represent the single deconvoluted curves, which highlight the different peaks contributing to the spectrum, and the red line represents the sum of the deconvoluted curves (i.e., the adjustment to the spectrum, whose goodness of fit expressed as *R*^2^ value). 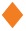 Signature peaks for pheomelanin, 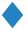 signature peaks for chitin, 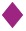 signature peaks for *N*-acetyl-d-glucosamine. (A) Orange hair on the abdomen of *Bombus mesomelas*; (B) orange hair on the thorax of *Bombus dahlbomii*; (C) red hair on the abdomen of *Bombus lapidarius lapidarius*; (D) red hair on the abdomen of *Bombus monticola*; (E) brown hair on the abdomen of *Bombus pascuorum dusmeti*; (F) brown hair on the thorax of *Bombus humilis*.

On the other hand, pheomelanin seemed to be the only predominant melanin in the yellow, orange, red and brown colors of the pubescence (Figs. 4 and 5). Indeed, the distinct peaks of its signature (1,490 and 2,000 cm^−1^) (confirmed by the reference deconvolution method) were well visible (Figs. 4 and 5), while no eumelanin-related peaks were detected.

Other peaks found in all Raman spectra, such as those at 1,445, 1,640 and 713 cm^−1^ (Figs. 1–5) were previously associated with the vibration modes of chitin, while three peaks at 514, 625 and 649 cm^−1^ are characteristic of the *N*-acetyl-d-glucosamine (De Gelder et al., 2007) (Figs. 1–5). Peaks associated with chitin (713 and 1,401 cm^−1^) and *N*-acetyl-d-glucosamine (1,315 and 1,564 cm^−1^) were also predominant in the spectrum obtained from hairless cuticle of the ventral side of *Bombus terrestris* thorax, where no melanin signatures could be detected (Fig. S2).

The Raman spectrum of the hair-extracted yellow pigment failed to reveal, as expected from its very low solubility, the pheomelanin signature (Fig. S3A); in addition, it did not reveal the typical strong peaks of pterin (687 and 1,309 cm^−1^), which were clearly visible in the spectrum obtained from synthetic pterin (Fig. S3B). Though both pheomelanin and pterin are composed of heterocycles and may thus have typical peaks in similar positions, actually one of the major peaks of pterin (1,309 cm^−1^) did not fall very close to the closest pheomelanin peak (at 1,490 cm^−1^). A high peak at ≈1,300 cm^−1^, was well visible in our spectra from yellow as well as from other colors, while Hines (2008a) excluded pterin occurrence in non-yellow hairs; this peak was very close to one of the typical peaks of chitin (1,315 cm^−1^), so it may not represent pterin. The other important peak for pterin (687 cm^−1^) also may fall under a high peak found in our spectra (≈700 cm^−1^), but, again, it is visible in spectra of all colors and is very close to the chitin-related peak at 713 cm^−1^.

**Figure 6:**
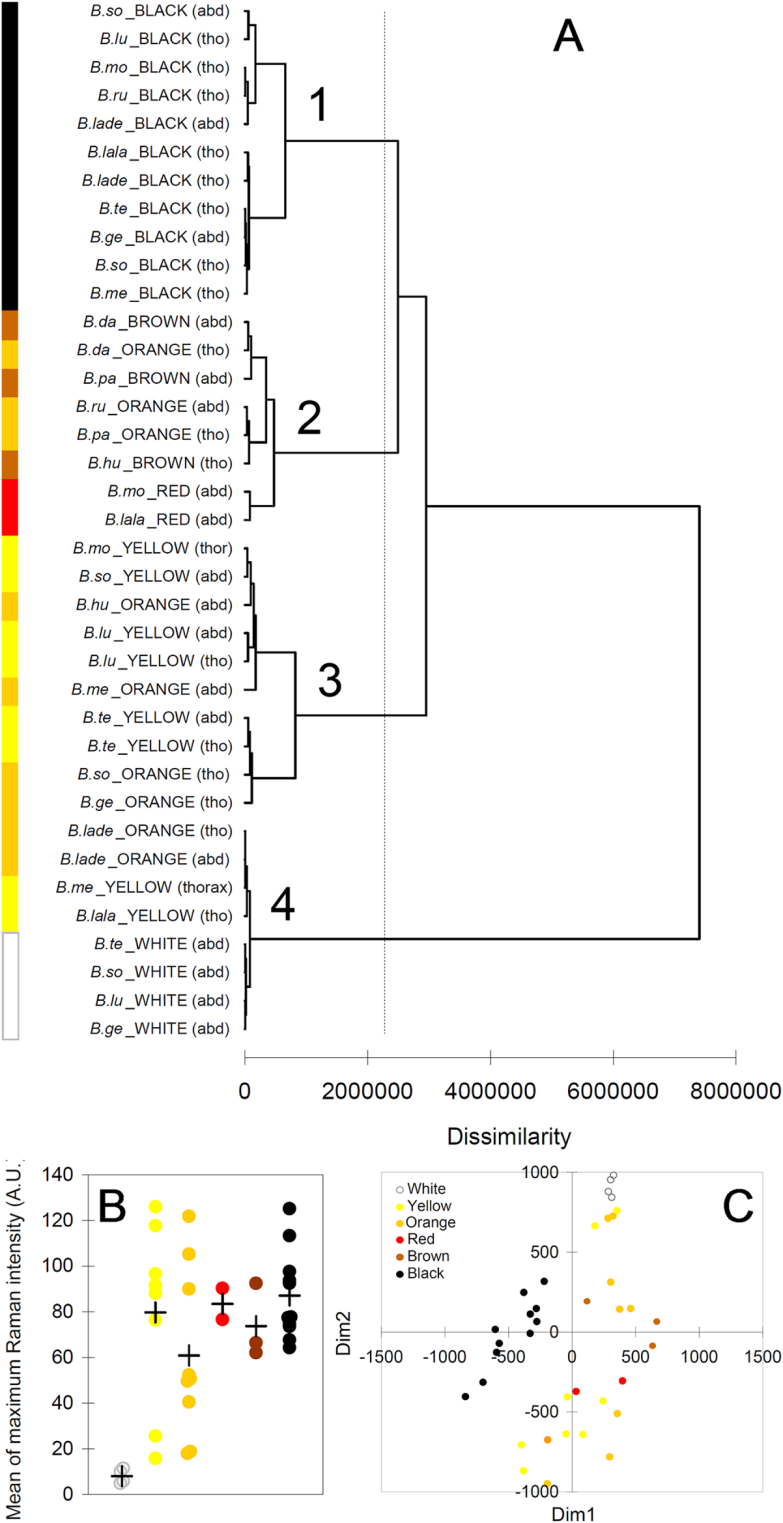
Results from multivariate analyses. (A) Dendrogram obtained from the agglomerative hierarchical clustering (dashed vertical line represents the dissimilarity value which discriminates the major different clusters); (B) relationship between colors and maximum intensity signals in the Raman spectra (+ symbols identify mean values across species/color/body part); (C) multi-dimensional scaling plot, based on the mean values of each peak across individuals of each species/color/body part. Species abbreviations in (A): *B.so*, *Bombus soroeensis*; *B.lu*, *Bombus lucorum*; *B.mo*, *Bombus monticola*; *B.ru*, *Bombus ruderarius*; *B.lade*, *Bombus lapidarius decipiens*; *B.lala*, *Bombus lapidarius lapidarius*; *B.te*, *Bombus terrestris*; *B.ge*, *Bombus gerstaerckeri*; *B.me*, *Bombus mesomelas*; *B.da*, *Bombus dahlbomii*; *B.pa*, *Bombus pascuorum dusmeti*; *B.hu*, *Bombus humilis.*

The cluster analysis (AHC) based on the Raman spectra reasonably agreed with the presence and with the type of melanin, as well as, to some extent, with the observed color (Fig. 6A). The first bifurcation of the dendrogram separated all white parts (no melanins) (group 4), together with few yellow–orange parts, from the rest of sample (Fig. 6A). These few yellow–orange spectra (four out of 19) falling close to the white spectra had especially poor signals. The melanin signature is visible, but the maximum signal is low (15–25 AU), closer to that in white spectra (4–11 AU) than to that in the other spectra (50–125 AU) (Fig. 6B). Within the remaining large group of the dendrogram, there was a tendency to separate yellow and orange parts (Fig. 5A, group 3) from darker colors, i.e., red, brown and black (Fig. 5A, group 2). Then, a further bifurcation discriminated all black parts (eumelanin + pheomelanin) (group 1) from the rest (pheomelanin only) (Fig. 5A). Body part (thorax or abdomen) did not seem to affect the distribution of cases in the dendrogram. For example, different black body parts were intermixed in group 1 (Fig. 6A). Similarly, phylogeny of the studied species did not seem to account for dissimilarity between cases. For example, spectra of *Bombus soroeensis*, *Bombus lucorum*, *Bombus terrestris* and *Bombus monticola* clustered in either group 1 or group 3 depending on being black or yellow (Fig. 6A).

The plot derived from the multidimensional scaling analysis (Fig. 6C) confirmed the pattern shown in the cluster analysis. Black body parts are concentrated in an area on the left (negative values of D1) of the plot, roughly around null D2; other dark colors (red and brown) seem also to remain in similar position respect to D2, but have positive D1 values. Lighter colors (yellow, orange) seem also to have mostly positive D1, but having mainly similar or higher values of D2 compared with darker colors. White body parts are strongly clustered at the upper limit of D2 in a characteristic position, together with the few low-signal spectra of yellow and orange.

Bumblebee hair color variation is thus predominantly due to both types of melanin (black), pheomelanin only (yellowish to brownish), or lack of pigmentation (white). Raman spectra even varied to some extent with the darkness degree of the observed colors, and not with phylogeny of the studied species.

## Discussion

Despite no previous analytical study was carried out to determine the association between colors and type of melanin in *Bombus*, our findings confirm the long-time suggested hypothesis that melanin is responsible for black and orange/red/brown coloration (Babiy, 1925; Hines, 2008a). While the association between black and eumelanin is not surprising, since this pigment was often reported in insects’ black body parts (Needham, 1978; Lindstedt et al., 2010; Galván et al., 2015), pheomelanin was reported in very few cases (Galván et al., 2015; Jorge García, Polidori & Nieves-Aldrey, 2016), and to date was only suspected for bumblebee reddish hairs (Hines, 2008a). These colorations have a relatively simple genetic basis (Owen & Plowright, 1980; Owen, Whidden & Plowright, 2010) and may result from changes made during the same developmental pathway (Hines, 2008a).

The fact that melanin biosynthesis is at the basis of all colors except white is especially interesting because future molecular investigations would allow comparisons with other species in which melanin pathways and genetic control of pigmentation were studied (Wittkopp, Carroll & Kopp, 2003; Lemonds, Liu & Popadić, 2016). These new studies may find a starting point from our work and by the recent description of the draft genomes of two bumblebee species (Sadd et al., 2015), which revealed the presence of the *dopa-decarboxylase* and *prophenoloxidase*, two genes involved in pigmentation/melanin synthesis in other insects (Koch et al., 1998; Zufelato et al., 2004).

Black, which we have showed is due a mixture of eumelanin and pheomelanin, is the most common pubescence color across bumblebee species (Rapti, Duennes & Cameron, 2014), suggesting that it may serve as a ground plan color which forms contrasts with other colors (and thus aposemantism). Interestingly, black hair pigmentation in bumblebees seems to be different from black cuticle in other insects and spiders, in which eumelanin is the only melanin type present (Galván et al., 2015; Hsiung, Blackledge & Shawkey, 2015; Jorge García, Polidori & Nieves-Aldrey, 2016). The fact that eumelanin and pheomelanin both occur in black hairs agrees with observations on the color’s change experienced by bumblebees during their adult life. For example, Friese & Wagner (1910) noted that bumblebees shift from gray–white hair (in callows) to either yellow or red, the latter in some cases further shifting to black. Gray colors in callow may be due to traces of both eumelanin and pheomelanin, and these shifts may be associated first to an increase of pheomelanin (in the route to yellow and red) and then to an increase of eumelanin (from red to black). Hines (2008a) showed a lack of fluorescent pigments in callow hairs fated to become yellow, thus also suggesting that only melanins (which do not fluoresce) at very low abundance occur in very young individuals.

Our Raman experiments were not sufficient to clearly associate yellow with a non-melanic pigment. In the past, the yellow in bumblebee hair was suggested to be due to either flavonoids (Stein, 1961) or pterins (Hines, 2008a). By TLC and HPLC/MS chemical analyses, Hines (2008a) discarded the presence of flavonoids and suggested that the yellow pigment is a small fluorescent heterocyclic compound, as a pterin, remarkably similar in properties across bumblebee lineages. Indeed, the yellow pigment shows the characteristic shifts in pH expected of pterins and its mass (177 mw) excludes the possibility of both flavonoids and melanins or melanin intermediates (which furthermore do not fluoresce as pterins do) (Hines, 2008a). However, in contrast with this finding, we obtained clear pheomelanin signature from yellow hairs, and no correspondence with the typical Raman spectra of synthetic pterin. In addition, our experiments showed that the Raman did not detect pternis in the hair-extracted yellow pigment, though this could be due to its low abundance. Thus, at the moment we can prove that pheomelanin also occurs in yellow hair, but we cannot either confirm or reject the possibility that pterin also occurs. New experiments with different techniques are necessary to solve this point.

Apart from melanins and the still unclear role of pterin, no other pigments seem to be involved in bumblebee hair coloration. Indeed, based on the TLC and HPLC/MS analytical results of Hines (2008a), no pigments are xanthopterins (which confers for example yellow in some butterflies’ wing scales and yellow cuticle in some social wasps (Wijnen, Leertouwer & Stavenga, 2007; Plotkin et al., 2009)) and certainly not leucopterins (which confer white, not yellow, coloration). Furthermore, available Raman spectra for xanthopterin revealed a high peak at ≈1,150 cm^−1^ that lacked in our spectra (Saenko et al., 2013). Carotenoids (having a very high peak at ≈1,150 cm^−1^ absent in our spectra) and ommochromes (having a very high peak at ≈1,800 cm^−1^ absent in our spectra), also found in arthropods (Nijhout, 1997; Heath, Cipollini & Stireman, 2013), can also be excluded by comparisons with available Raman spectra (Merlin, 1985; Hsiung, Blackledge & Shawkey, 2015) and following Hines (2008a).

Our results seem to support previous considerations arisen from the study of color pattern diversity and frequency. Rapti, Duennes & Cameron (2014) found that various orange and yellow colors occur at high frequency in the abdomen, suggesting that different pigment classes may be derived from the same pigment that varies in density within the setae. This pigment was unknown at the time of this consideration, and we now show that it is pheomelanin. In addition, changes from yellow to black or black to yellow are among the most common changes in color (Rapti, Duennes & Cameron, 2014). This now makes sense given that pheomelanin is present in both black (together with eumelanin) and yellow hair; thus, a decrease or disappearance of eumelanin in black hair could turn hair to brighter colors. This does not contrast with the possibility that, particularly in yellow hairs, pheomelanin co-occurs with a non-melanin pigment (Hines, 2008a). Pheomelanin would thus lead to all the other colors except white by varying in intensity and different modes in frequency of the stretching vibration of the hexagonal aromatic rings, symmetric and asymmetric tensions, in- and out-of-plan deformations and other vibration characteristics (Socrates, 2004; I. Galván et al., 2016, unpublished data), which affect how the pigment interact with radiation and in turn affect the pigment’s optical properties. Overall, it would be thus not necessary for bumblebees to produce a wide range of pigments to diversify their color patterns. The use of both melanin types seemed to be ancient in *Bombus*, when looking at the more recent phylogeny of the genus (Fig. 1 and Cameron, Hines & Williams, 2007). This probably allowed bumblebees to promptly diversify their color patterns during the genus radiation (Hines, 2008b; Rapti, Duennes & Cameron, 2014).

A very different situation occurs with white color, which was clearly associated with a lack of melanins in our study. The very noisy and weak spectra obtained from white hairs, with no clear peaks, did not point toward the presence of any other types of pigments (at least within the Raman detection power). A similar result was obtained for white body parts in spiders (Hsiung, Blackledge & Shawkey, 2015). In other cases, such as in *Bombyx mori*, white is probably due to uric acid and pteridine (Okamoto et al., 2008). In our case, the spectra from white hair resemble the spectra from hairless ventral side of the thorax, where only chitin-related peaks are visible. Though we did not find signature of eumelanin in the hairless black cuticle in *Bombus*, this pigment is, however, likely to occur, as melanins are known to be responsible for dark coloration in insects in general (Needham, 1978); however, eumelanin is probably located too deep within the cuticle matrix to be detected by Raman. Based on her TLC and HPLC/MS analytical results, Hines (2008a) also raised the possibility that white hairs are depigmented, but did not discard the possibility that small amounts of the non-melanic pigment of yellow hair occur, a hypothesis that needs further experiments to be tested.

The provided evidence that both eumelanin and pheomelanin are predominant in bumblebee hair opens to new studies in which concentrations of these pigments could be measured in individuals living in different environments or reared under different laboratory experiments, in order to link melanization, temperature and oxidative stress (which is linked with pheomelanin production (Galván et al., 2015; Napolitano et al., 2014)). This may help understanding the observed, and still not fully understood, variations of bumblebee color patterns along latitudinal gradients (Pekkarinen, 1979; Williams, 2007).

## Supplemental Information

10.7717/peerj.3300/supp-1 Supplemental Information 1 Patterns of coloration in queens and workers of *Bombus* species. (A–B) *B. lapidarius lapidarius* queen (dorsal-lateral) (C–D) *B. lapidarius lapidarius* worker (dorsal-lateral) (E–F) *B. humilis*_queen (dorsal-lateral) (G–H) *B. humilis*_worker (dorsal-lateral) (I–J) *B. lapidarius decipiens*_queen (dorsal-lateral) (K–L) *B. lapidarius decipiens_*worker (dorsal-lateral) (M–N) *B. ruderarius* queen (dorsal-lateral) (O–P) *B. ruderarius* worker (dorsal-lateral).

10.7717/peerj.3300/supp-2Supplemental Information 2Raman spectrum of hairless, black ventral side of thorax in *B. terrestris*, and peaks identification after having applied the Reference Deconvolution Method.The grey line represents the Raman spectrum, the dashed green lines represent the single deconvoluted curves, which highlight the different peaks contributing to the spectrum, and the red line represents the sum of the deconvoluted curves (i.e. the adjustment to the spectrum, whose goodness of fit expressed as R^2^ value). 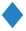 signature peaks for chitin, 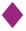 signature peaks for *N*-acetyl-d-glucosamine. Note that no melanin peaks were detected in hairless cuticle.

10.7717/peerj.3300/supp-3 Supplemental Information 3 Raman spectra of yellow hair extracts and synthetic pterin. (A) yellow hair extracts from *B. terrestris* (c) and associated solvents (a, acidified methanol; b, sodium carbonate), and (B) synthetic pterin (black line) and yellow hair extracts (blue line). ♦ signature peaks for pterin. Note that yellow hair extracts did not return a spectrum with neither visible pheomelanin signature nor visible pterin signature.

10.7717/peerj.3300/supp-4 Supplemental Information 4 Raman spectra obtained from all tested individuals of Bombus. Each sheet includes the data for one specific hair color, while the last sheet includes the data for the hairless ventral side of the thorax of *B. terrestris*. The alternate white and grey columns in the six color-related sheets indicate different individuals.

## Additional Information and Declarations

### Field Study Permissions

The following information was supplied relating to field study approvals (i.e., approving body and any reference numbers): Field collections were approved by the Ministerio de Medio Ambiente y Medio Rural y Marino (Spain) (permit number: CO/09/106/2010), Comunidad de Madrid (Spain) (permit number: 10/091686.9/15), Junta de Castilla y León (Spain) (permit number: EP/CYL/366/2013), Gobierno de Aragón (Spain) (permit number: CSVM5-1XA8S-75HA5-OUREG), Gobierno de Navarra (granted on 25/05/2009) and Estación Cientifica de Huinay (Chile) (granted on 18/10/2014).

### Data Availability

The following information was supplied regarding data availability: The raw data has been supplied as Supplemental Dataset Files.
