# Supplementary figures and images for: Eumelanin and pheomelanin are predominant pigments in bumblebee (Apidae: Bombus) pubescence
Source: PeerJ. 2017 May 24;5:e3300. doi: 10.7717/peerj.3300 (PMC5445944; doi:10.7717/peerj.3300)

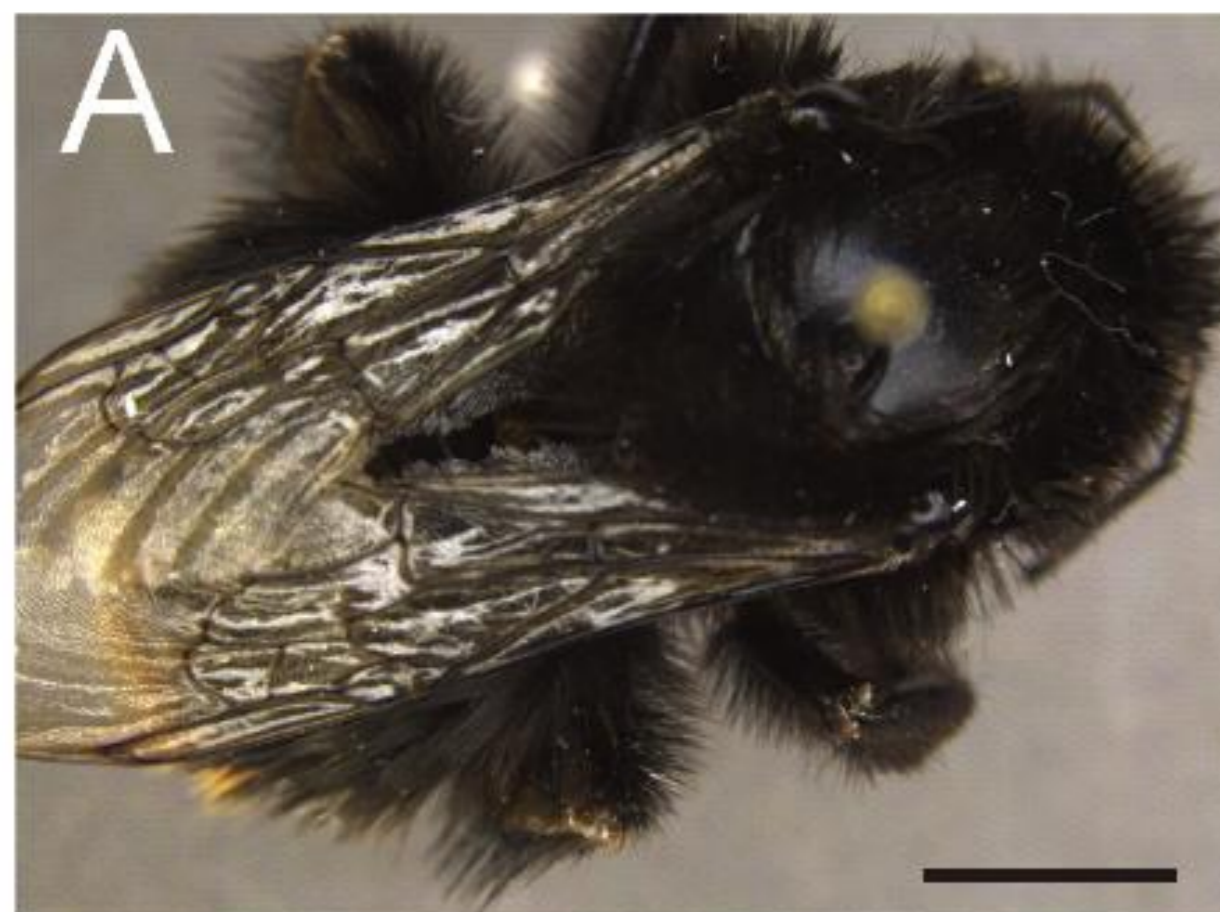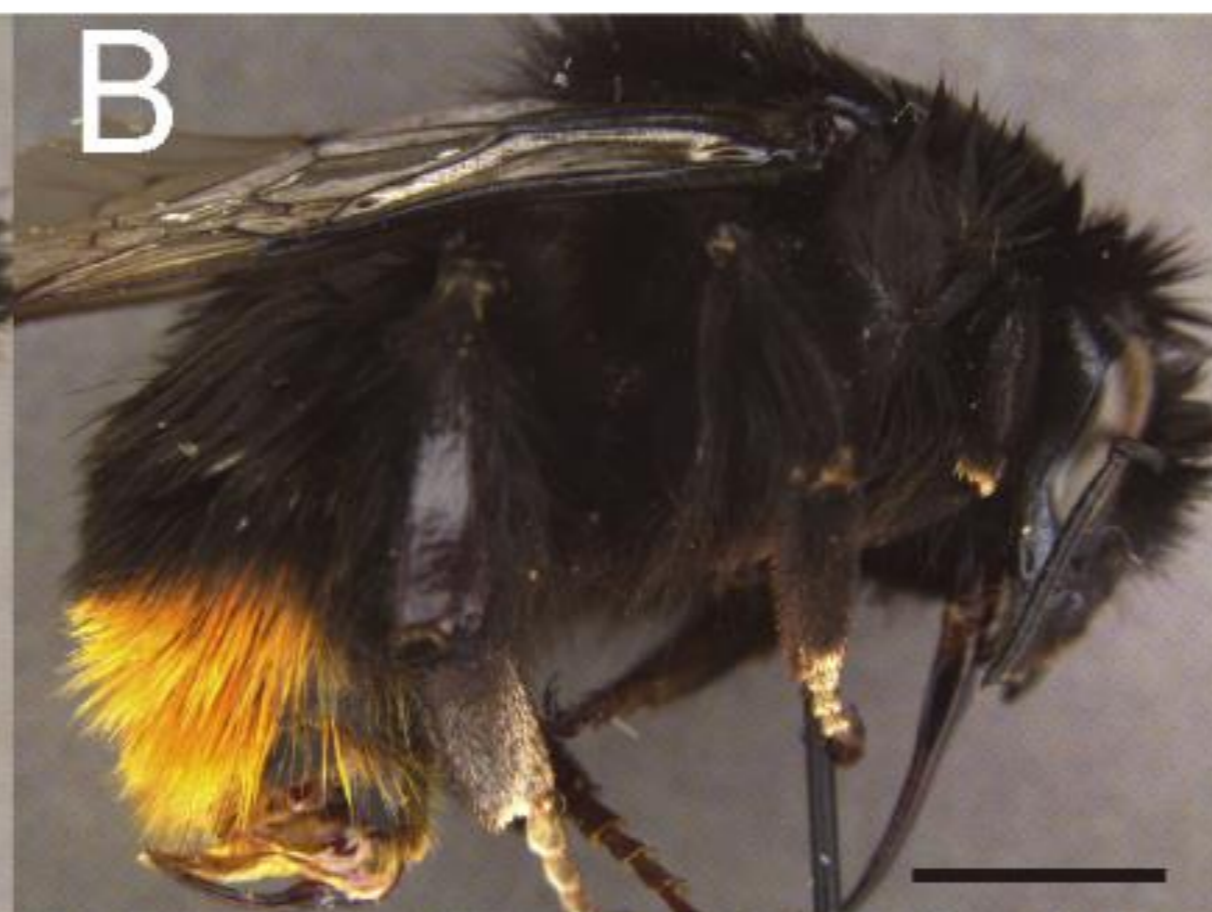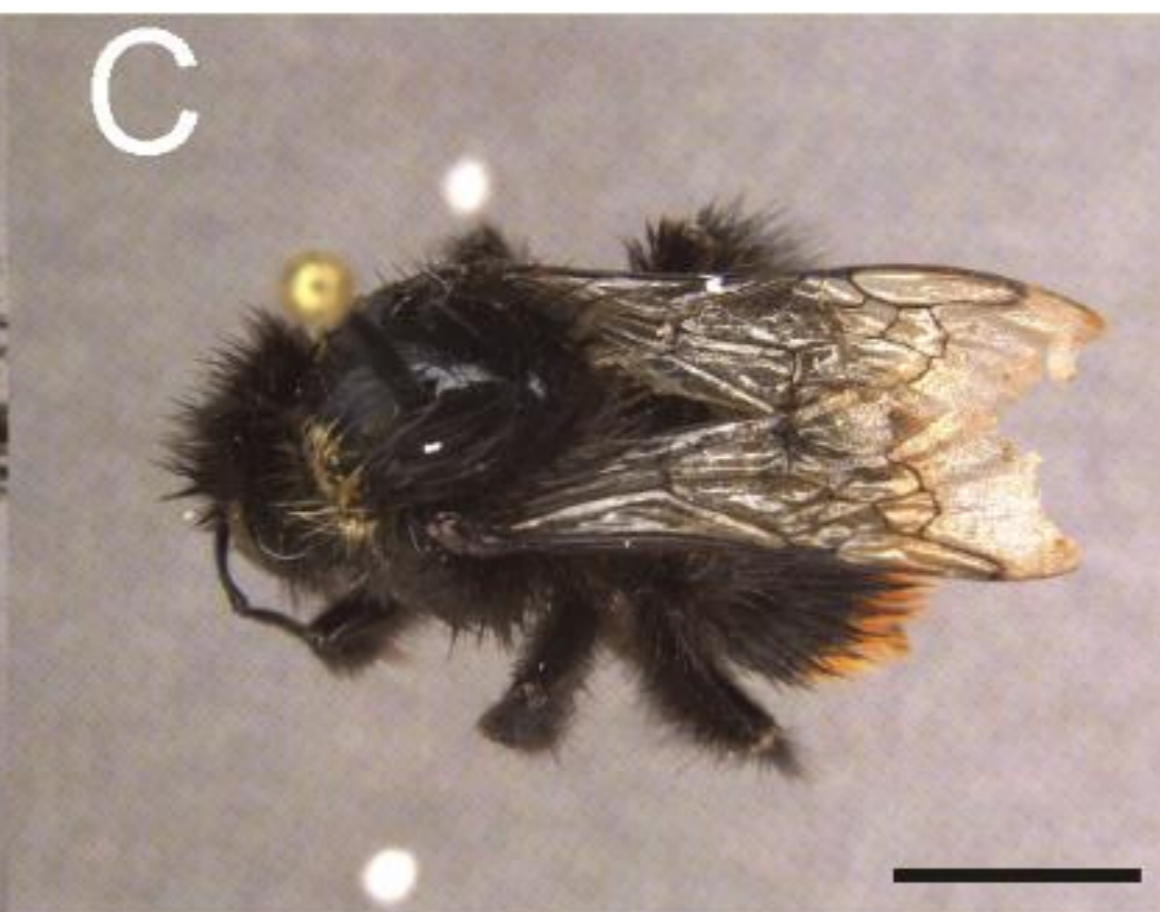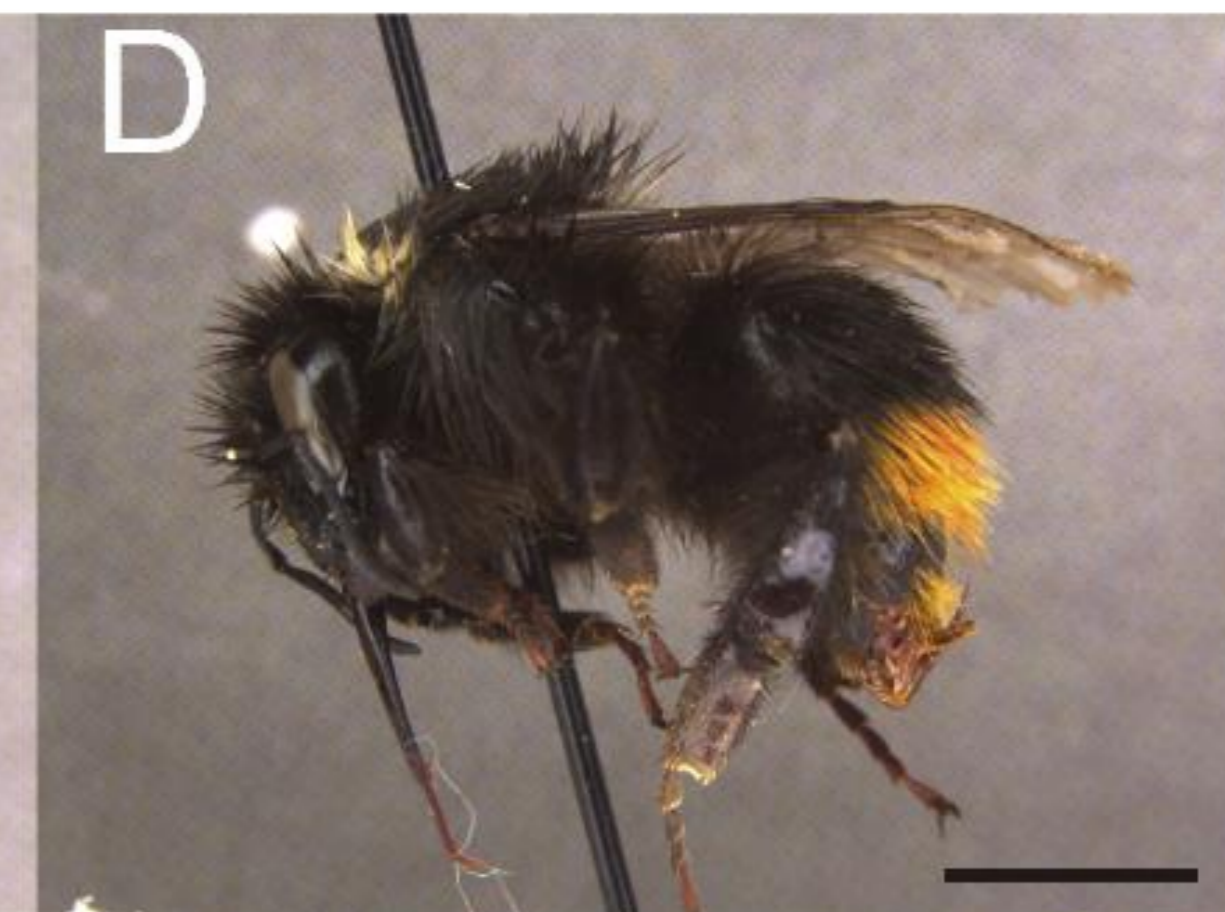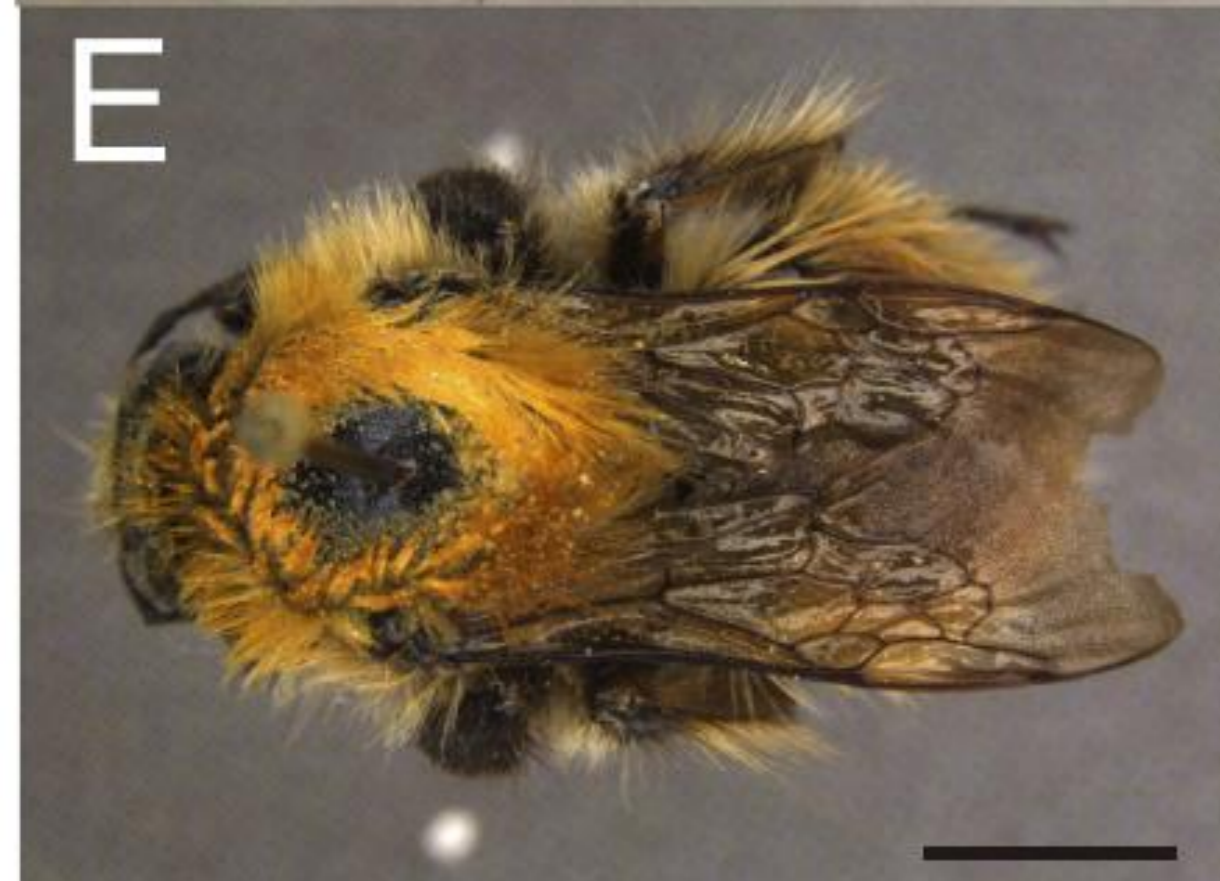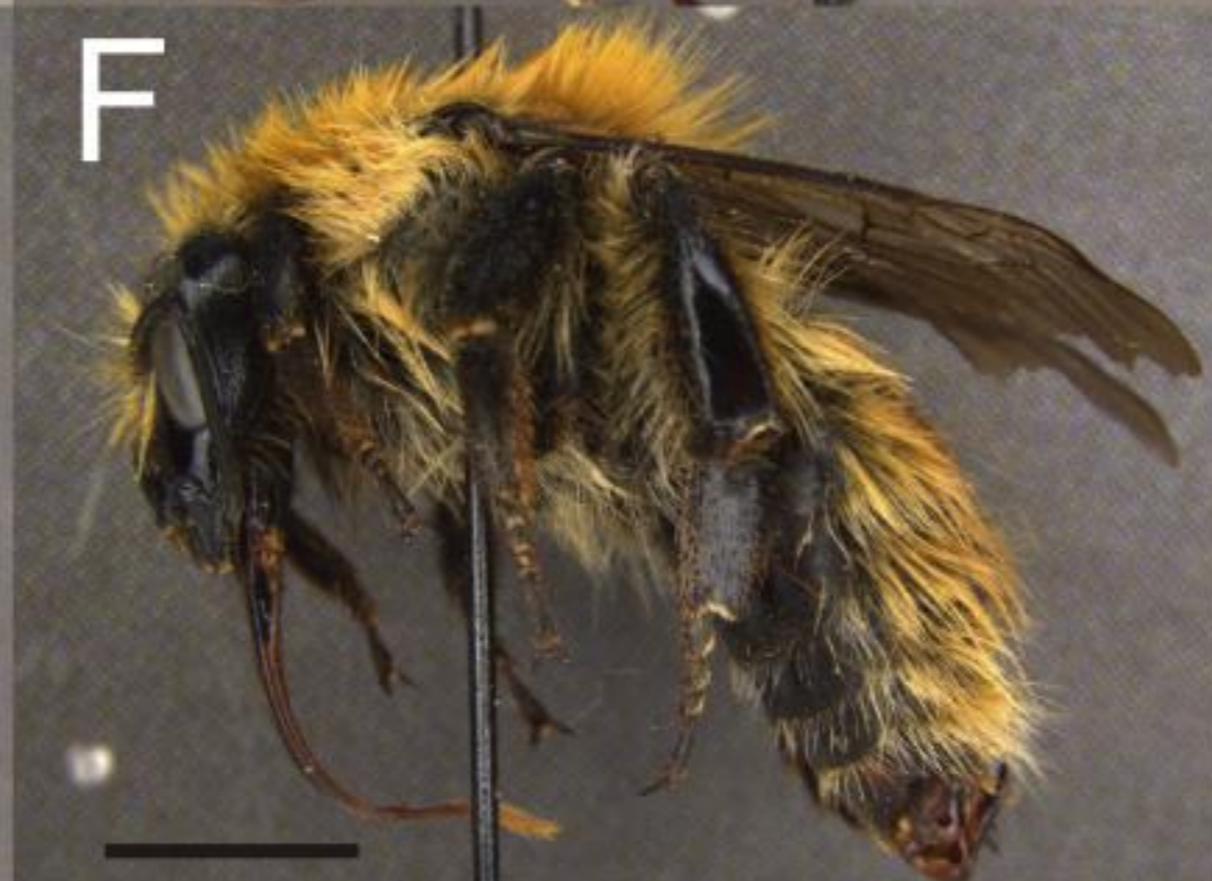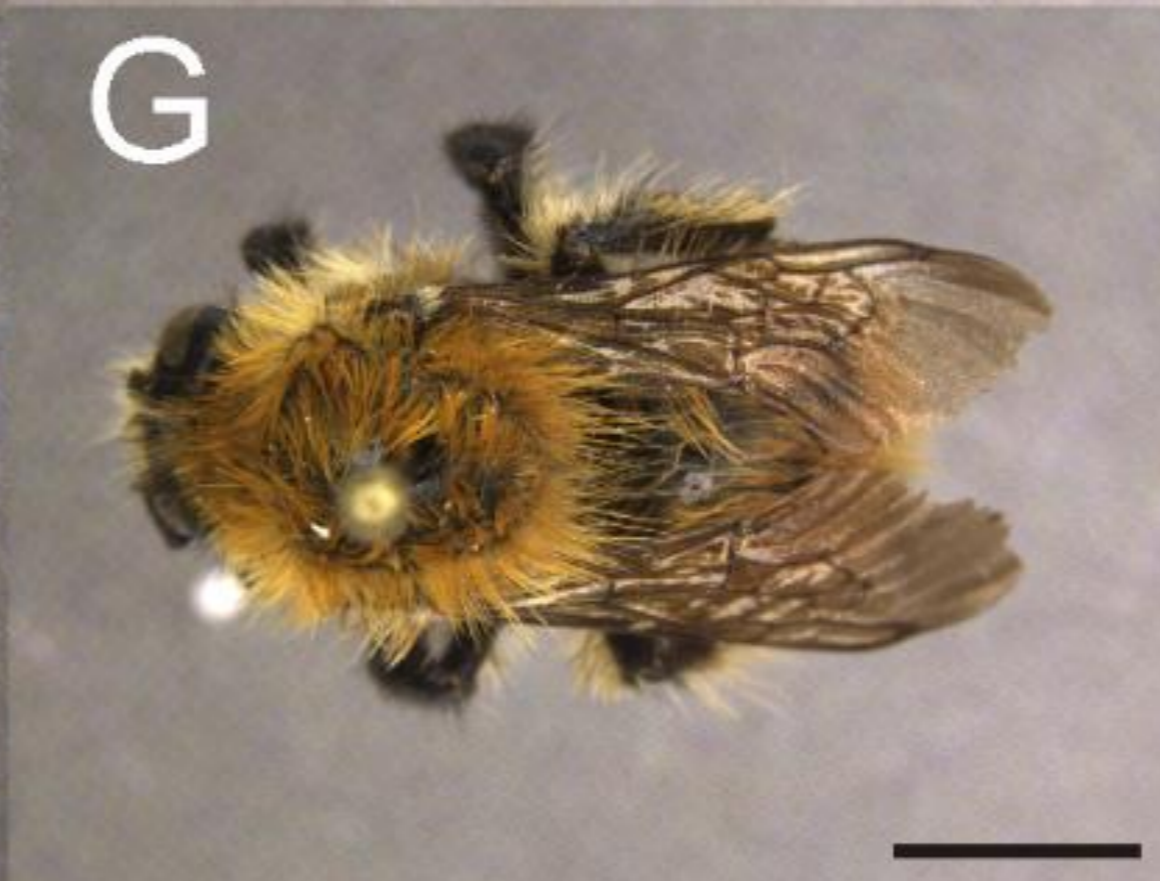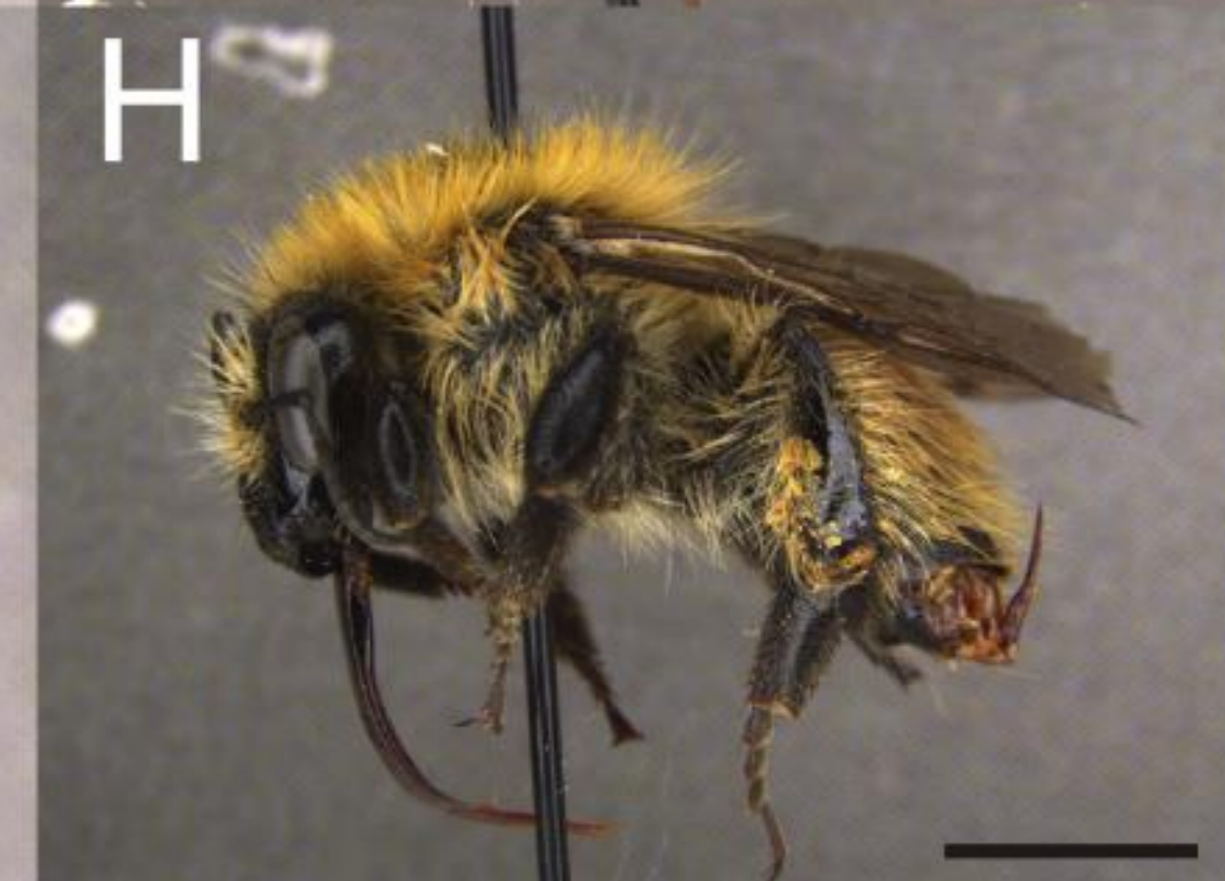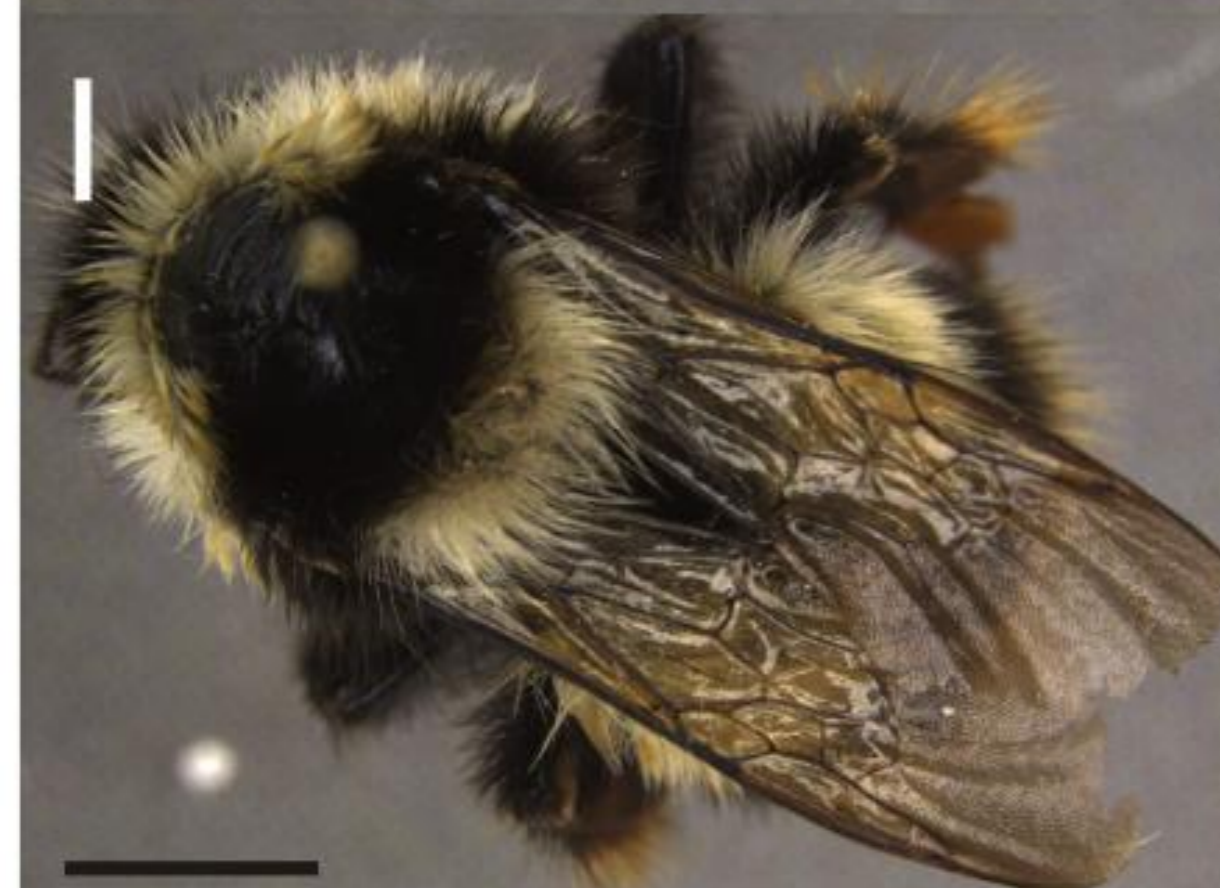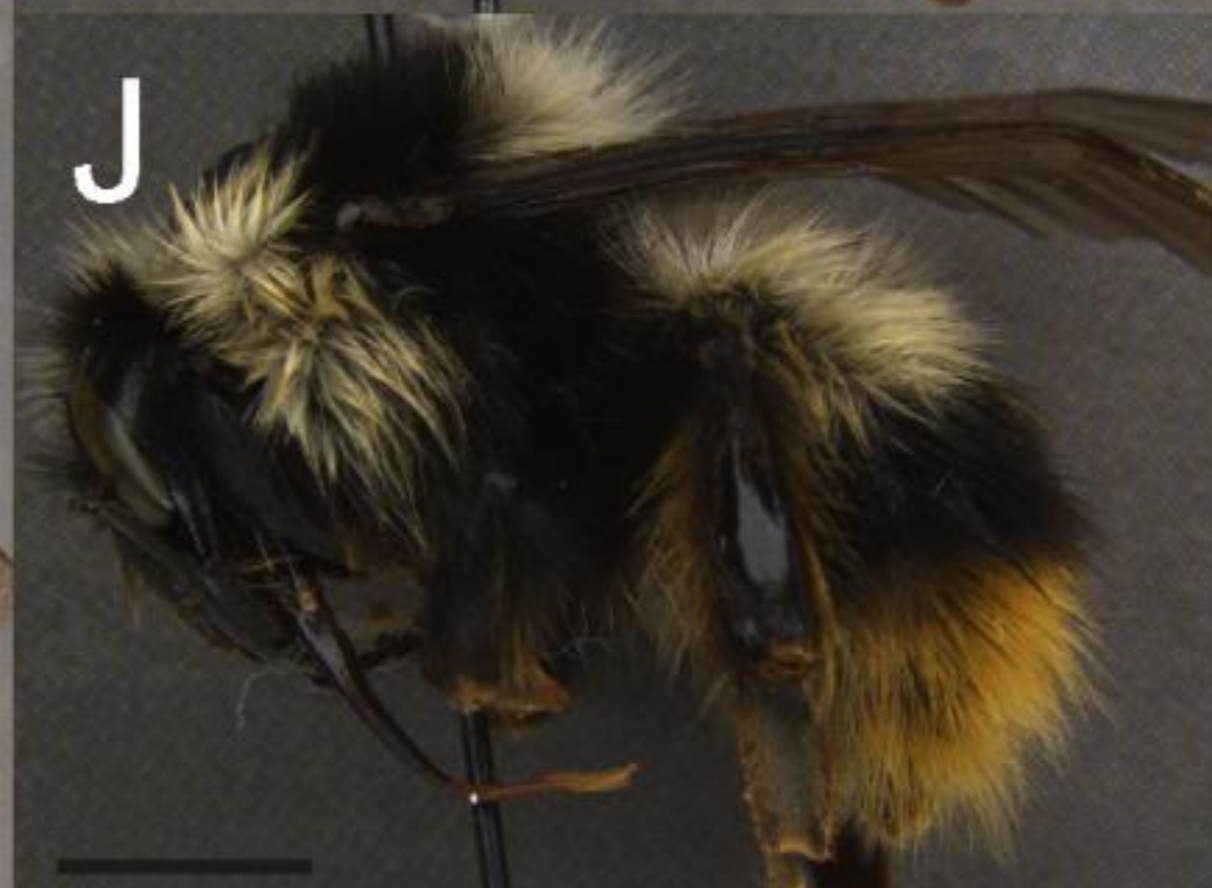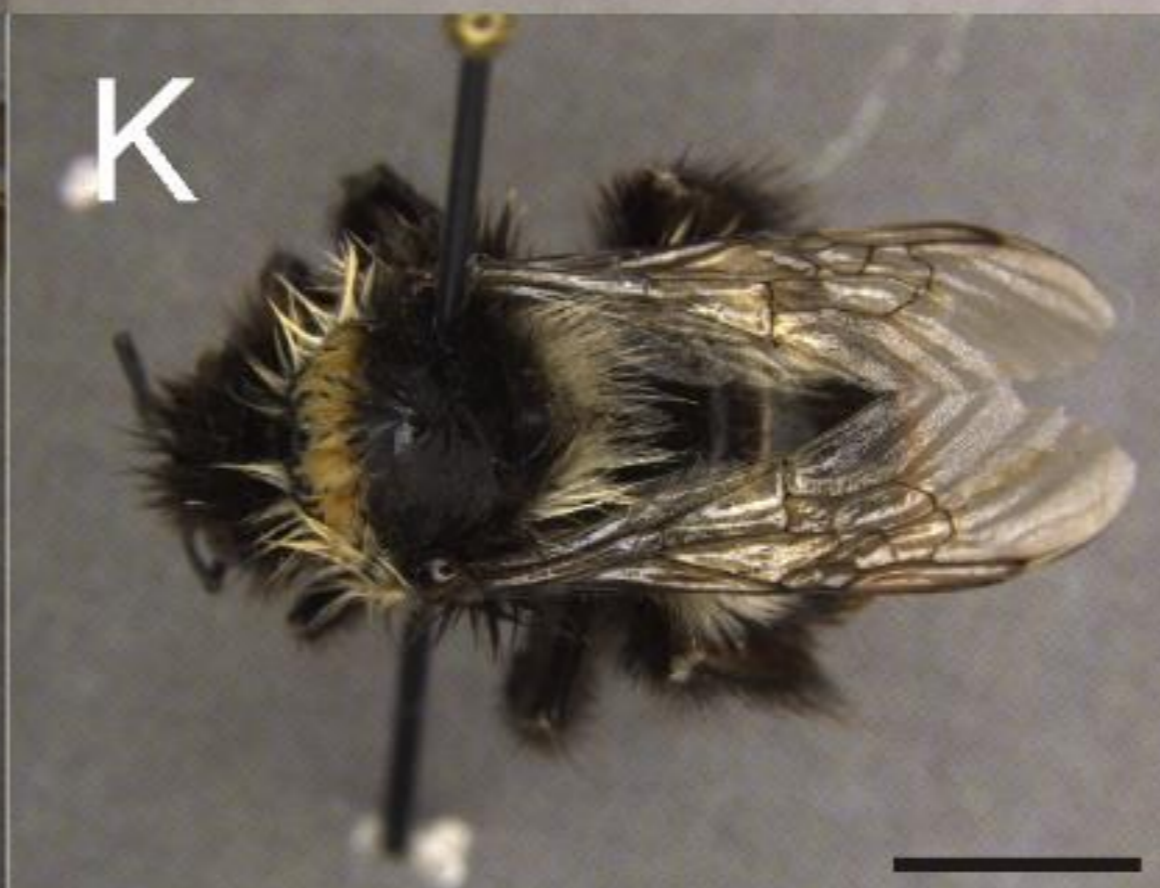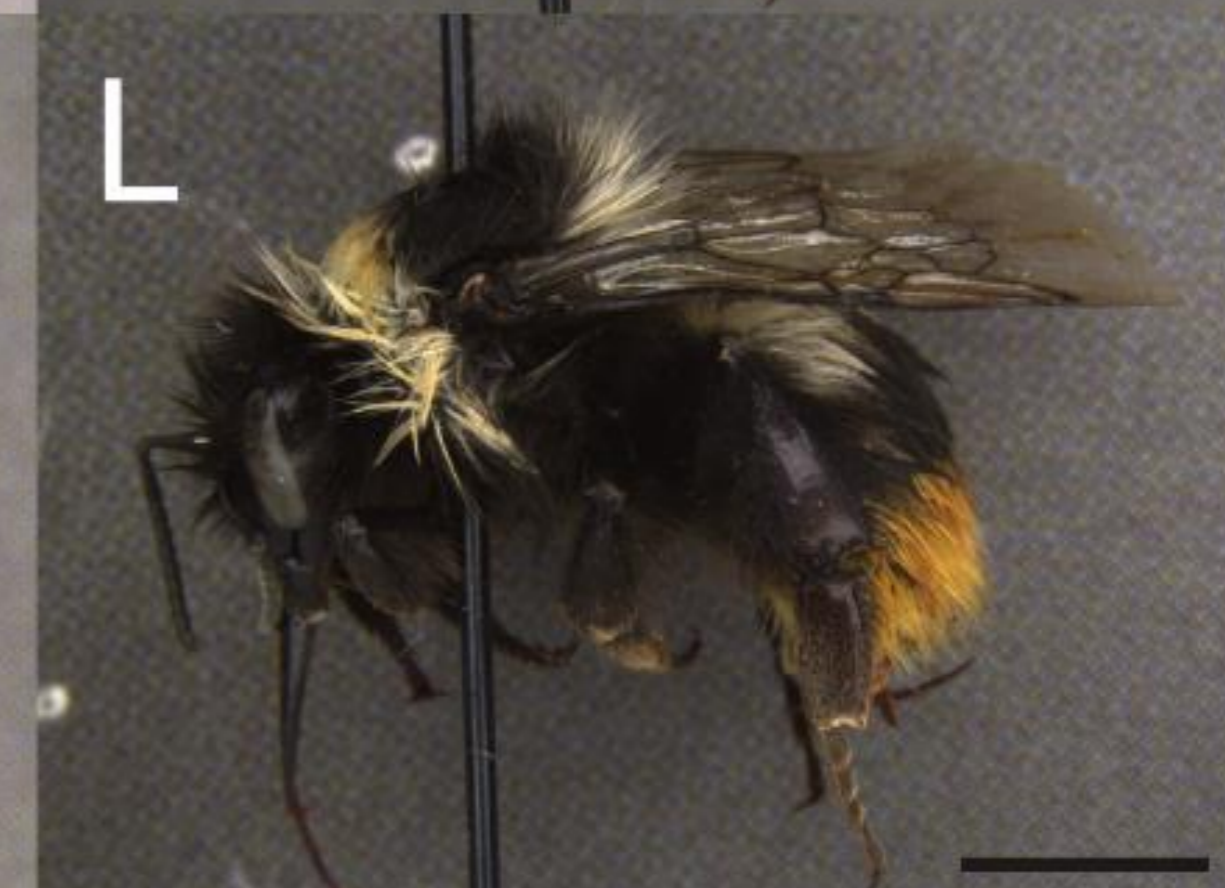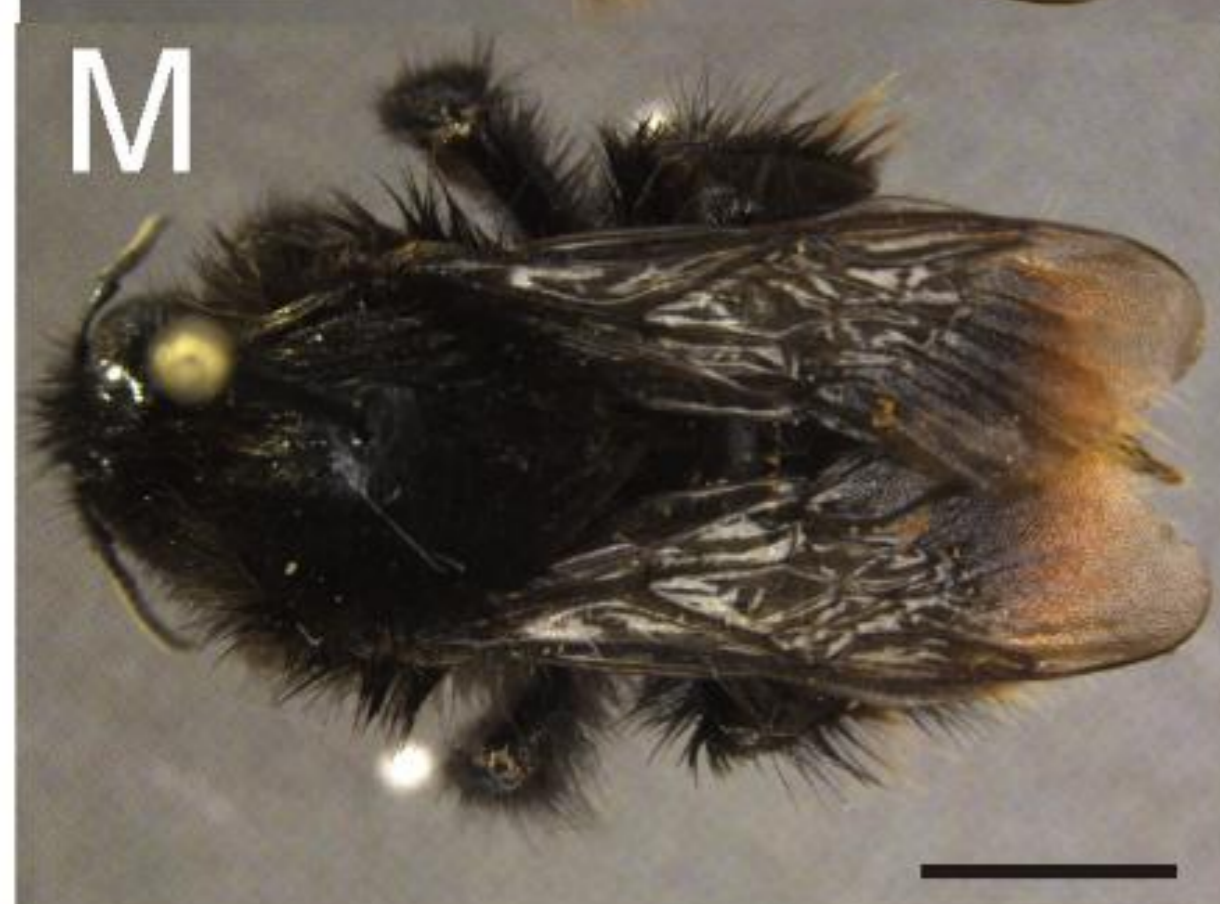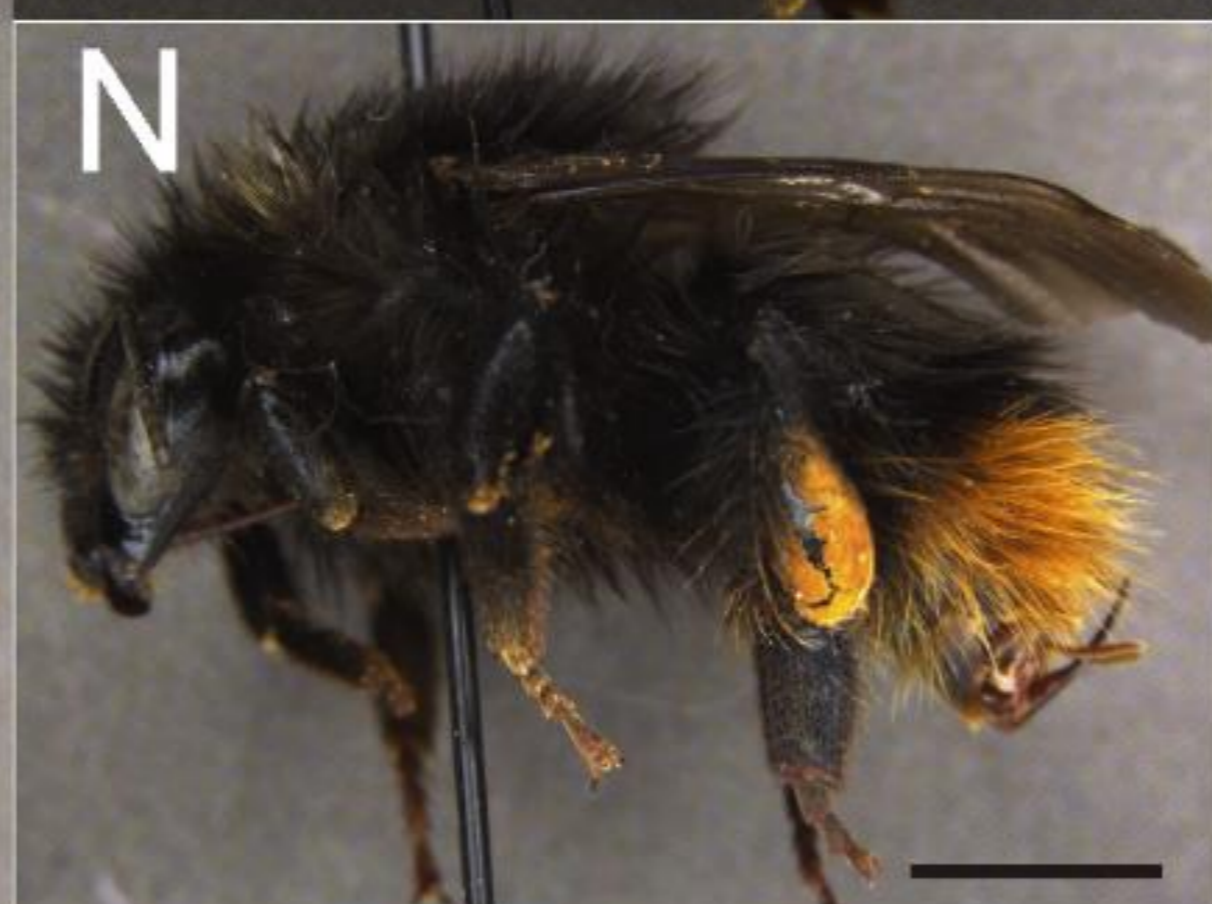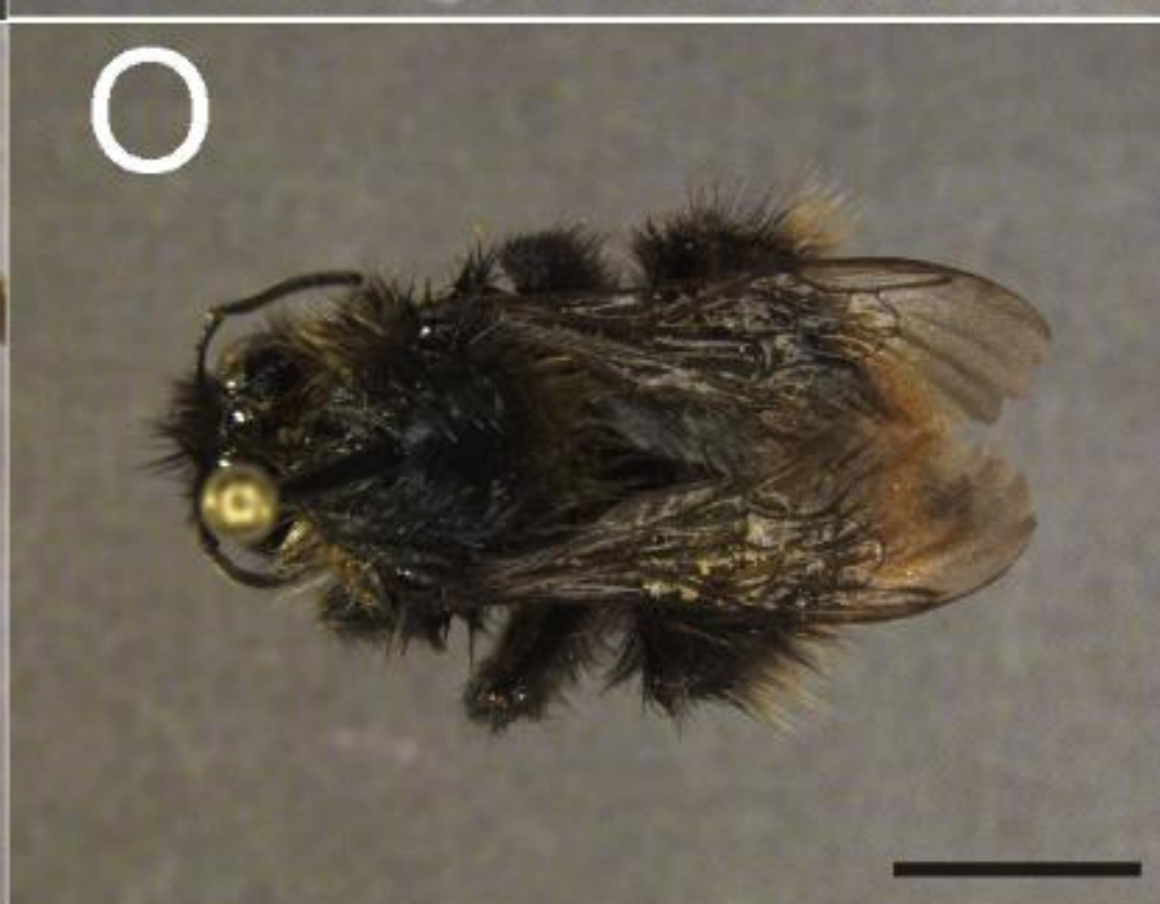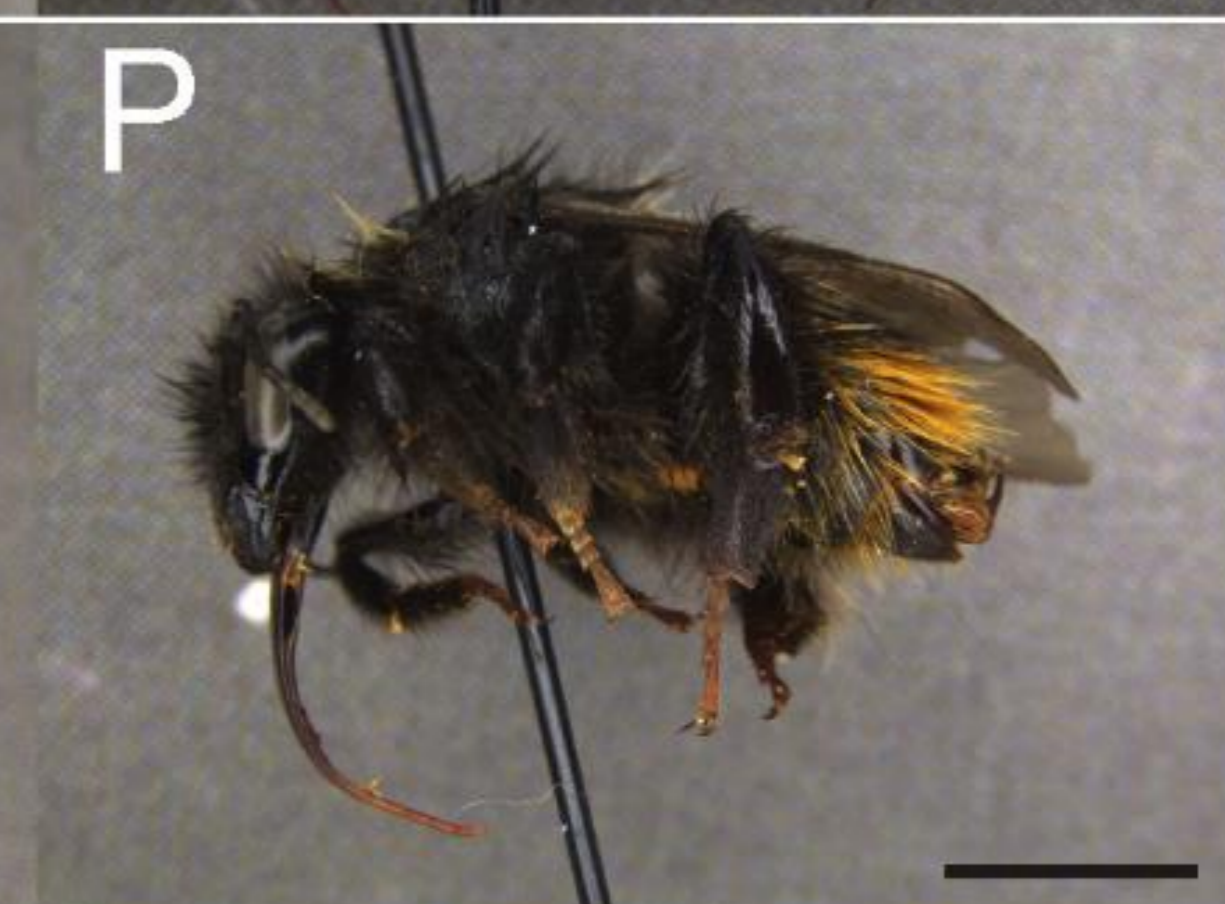

Supplement: Supplemental Information 1 — (A–B) B. lapidarius lapidarius queen (dorsal-lateral) (C–D) B. lapidarius lapidarius worker (dorsal-lateral) (E–F) B. humilis_queen (dorsal-lateral) (G–H) B. humilis_worker (dorsal-lateral) (I–J) B. lapidarius decipiens_queen (dorsal-lateral) (K–L) B. lapidarius decipiens_worker (dorsal-lateral) (M–N) B. ruderarius queen (dorsal-lateral) (O–P) B. ruderarius worker (dorsal-lateral). [file peerj-05-3300-s001.pdf]

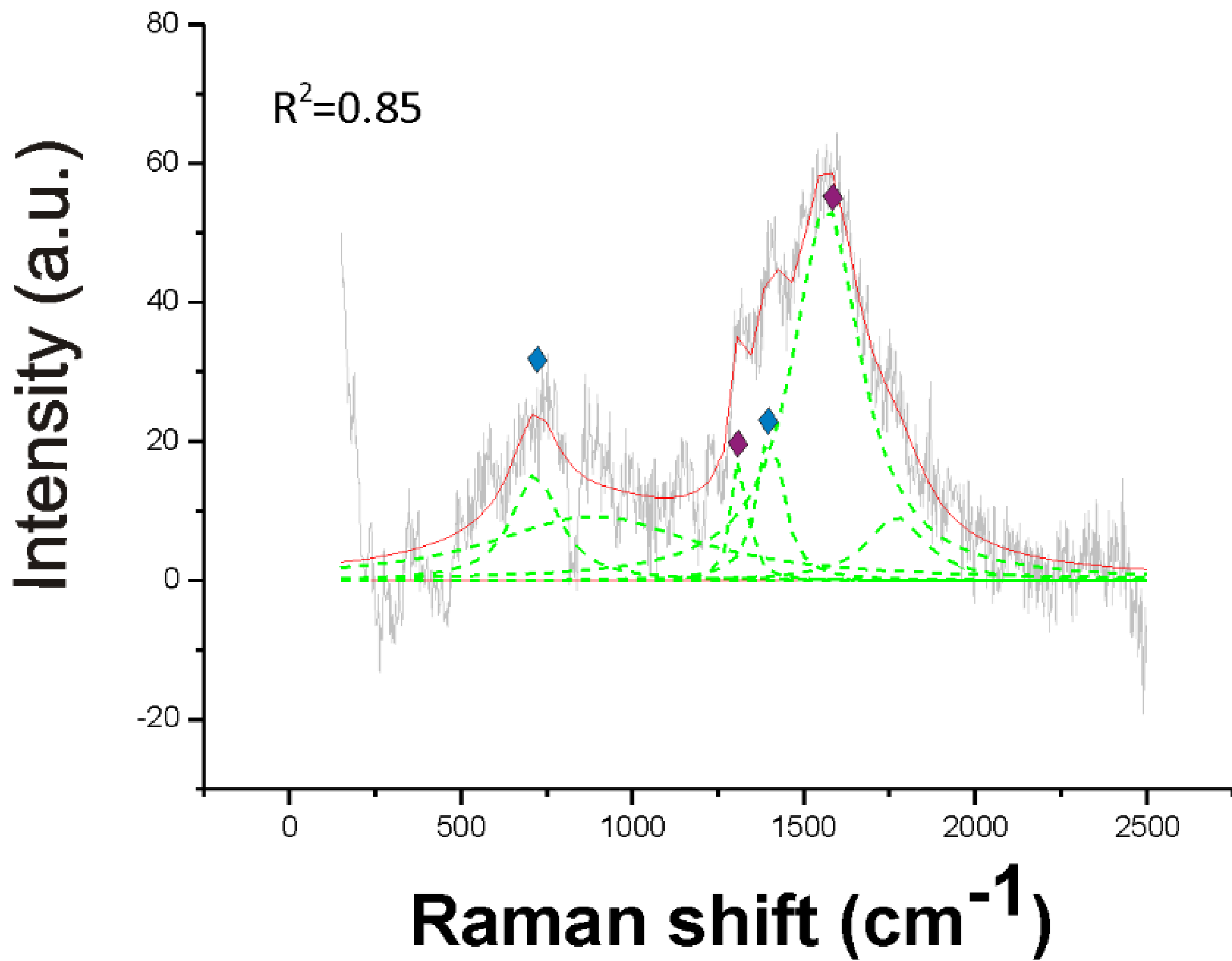

Supplement: Supplemental Information 2 — The grey line represents the Raman spectrum, the dashed green lines represent the single deconvoluted curves, which highlight the different peaks contributing to the spectrum, and the red line represents the sum of the deconvoluted curves (i.e. the adjustment to the spectrum, whose goodness of fit expressed as R2 value). signature peaks for chitin, signature peaks for N-acetyl-d-glucosamine. Note that no melanin peaks were detected in hairless cuticle. [file peerj-05-3300-s002.pdf]

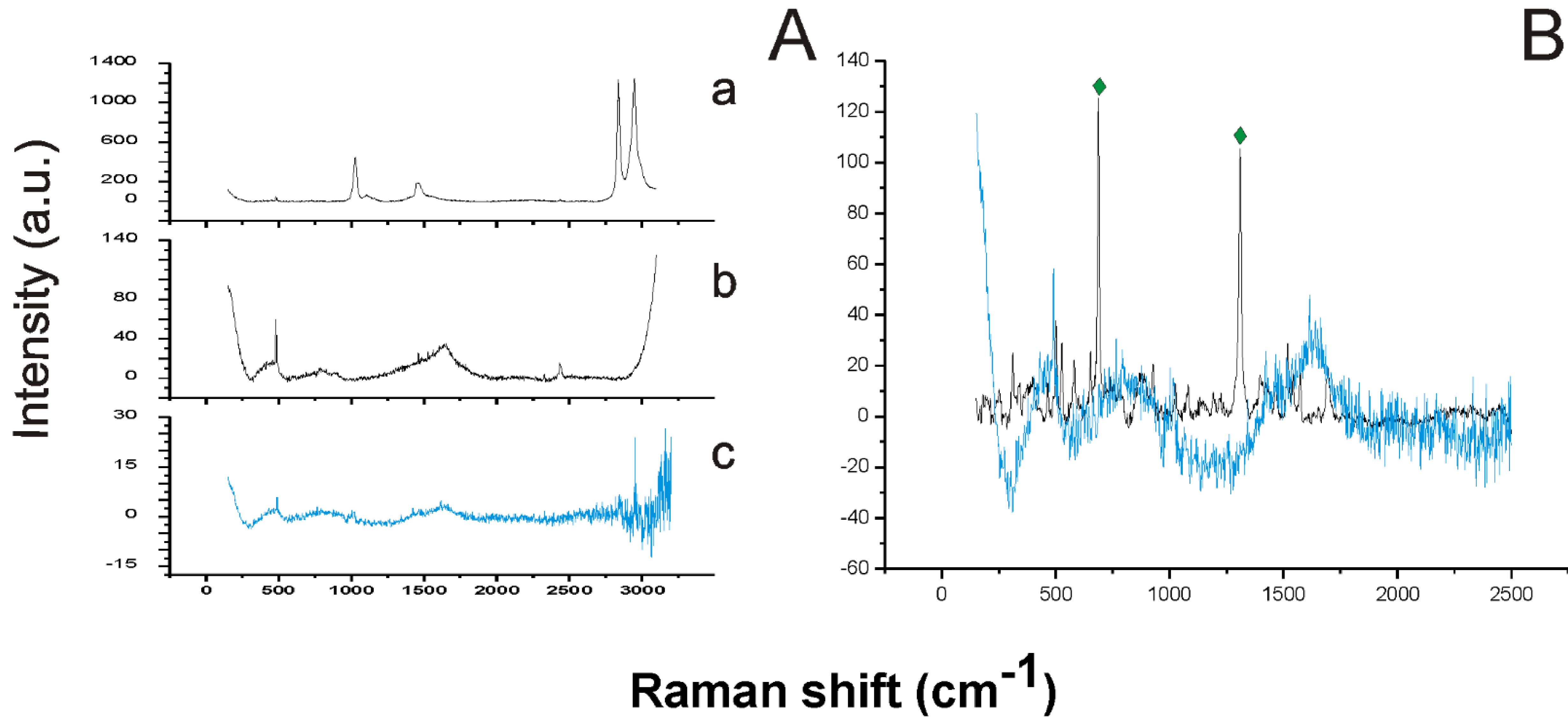

Supplement: Supplemental Information 3 — (A) yellow hair extracts from B. terrestris (c) and associated solvents (a, acidified methanol; b, sodium carbonate), and (B) synthetic pterin (black line) and yellow hair extracts (blue line). ♦ signature peaks for pterin. Note that yellow hair extracts did not return a spectrum with neither visible pheomelanin signature nor visible pterin signature. [file peerj-05-3300-s003.pdf]
